# Ampere‐Level Electrosynthesis of CO via Well‐Defined Pyridinic‐N Incorporated Cobalt Phthalocyanine

**DOI:** 10.1002/smll.202507824

**Published:** 2025-09-30

**Authors:** Tengyi Liu, Xiaofan Hou, Di Zhang, Yutaro Hirai, Kosuke Ishibashi, Yasutaka Matsuo, Junya Yoshida, Shimpei Ono, Hao Li, Hiroshi Yabu

**Affiliations:** ^1^ Advanced Institute for Materials Research (WPI‐AIMR) Tohoku University 2‐1‐1 Katahira, Aoba‐Ku Sendai 980–8577 Japan; ^2^ AZUL Energy, Inc. Sendai 980‐0811 Japan; ^3^ AZUL Energy & Tohoku University Co‐Creation Center Tohoku University 2‐1‐1 Katahira, Aoba‐Ku Sendai 980–8577 Japan; ^4^ Research Institute for Electronic Science (RIES) Hokkaido University N21W10, Kita‐Ward Sapporo 001–0021 Japan; ^5^ International Center for Synchrotron Radiation Innovation Smart Tohoku University Sendai 980–8579 Japan

**Keywords:** cobalt tetra‐aza‐phthalocyanine, electrochemical CO_2_ reduction, incorporation modification, pyridinic nitrogen

## Abstract

High‐rate CO electrosynthesis from CO_2_ is vital for efficient CO_2_‐CO‐C_2+_ tandem conversion. Cobalt phthalocyanine (CoPc), featuring a Co‐N_4_ site naturally favorable for CO production, suffers from low conductivity. Herein, a molecular engineering strategy is reported to construct cobalt tetra‐aza‐phthalocyanine (CoTAP) by incorporating four pyridinic‐N atoms at the β‐positions of the CoPc macrocyclic backbone, effectively enhancing both conductivity and intrinsic activity. The resulting CoTAP electrode achieves ≈100% CO selectivity at an ultralow onset overpotential of 140 mV (−0.25 V vs. RHE), significantly outperforming pristine CoPc (−0.57 V vs. RHE). Furthermore, it also delivers a record‐high CO current density of −1084 mA cm^−2^, an exceptional mass activity of 24,636.4 A g^−1^, and an ultrahigh turnover frequency of 73.4 s^−1^, with excellent stability for 112 h at −150 mA cm^−2^, surpassing all reported Pc‐based catalysts. Systematic analysis shows that pyridinic‐N incorporation alters the electronic environment around Co centers and reduces resistance to only 3.8% of CoPc. Theoretical calculations further confirm more favorable adsorption energies for key intermediates (^*^COOH and ^*^CO), underpinning the enhanced intrinsic activity. Collectively, these advancements maximize site‐specific reaction kinetics in CoTAP. This work presents a molecular‐level strategy to simultaneously boost conductivity and intrinsic activity for advanced CO_2_ electroreduction.

## Graphical abstract

Cobalt tetra‐aza‐phthalocyanine (CoTAP), incorporating four pyridinic‐N atoms at β‐positions of CoPc backbone, exhibits an ultralow onset potential (−0.25 V vs. RHE), a record *J*_CO_ (−1084 mA cm^−2^), exceptional mass activity (24636 A g^−1^), and excellent stability (112 h@−150mA cm^−2^), surpassing all reported Pc‐based‐catalysts. Systematic analysis and theoretical calculation reveal that pyridinic‐N incorporation markedly boosts both conductivity and intrinsic activity, outperforming pristine CoPc.

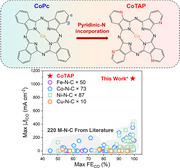

## 1 Introduction

The widespread reliance on fossil fuels has driven a steady rise in atmospheric carbon dioxide (CO_2_) levels, accelerating global warming.^[^ ^1^, ^2^, ^3^ ^]^ While renewable energy sources such as solar and wind are gaining momentum, their inherent intermittency poses significant challenges for efficient energy storage and utilization.^[^ ^4^, ^5^, ^6^, ^7^ ^]^ Electrochemical CO_2_ reduction (ECR) offers a mild and sustainable approach to upcycle CO_2_, especially when powered by renewable electricity, into energy‐dense, value‐added fuels and chemicals, presenting a promising strategy to reduce carbon emissions and advance carbon neutrality.^[^ ^4^, ^5^, ^8^, ^9^, ^10^, ^11^ ^]^ Among the ECR products, multicarbon (C_2+_) compounds are especially valuable due to their higher economic significance. The key step in forming C_2+_ products is C─C coupling, where copper‐based catalysts excel.^[^ ^12^, ^13^, ^14^ ^]^ However, this strength also limits their efficiency in the initial CO_2_‐to‐CO conversion.^[^ ^14^, ^15^ ^]^ Therefore, the development of highly efficient CO‐producing catalysts is critical for enabling tandem pathways and ultimately achieving efficient C_2+_ synthesis.^[^ ^14^, ^16^, ^17^ ^]^

Pc‐family catalysts with single‐atom active sites inherently exhibit excellent selectivity for CO_2_‐to‐CO conversion, making them attractive candidates for CO electrosynthesis.^[^ ^18^, ^19^, ^20^, ^21^ ^]^ For instance, our previous work identified cobalt phthalocyanine (CoPc) as a highly active and CO‐selective catalyst.^[^ ^22^, ^23^ ^]^ However, conventional Pc‐materials’ semiconductive nature and poor conductivity often hinder their further applications. A common strategy to address this issue involves combining Pcs with conductive carbon materials.^[^ ^20^, ^24^ ^]^ Achieving molecular‐level dispersion on these supports can mitigate problems such as molecular aggregation and low catalyst utilization.^[^ ^25^, ^26^ ^]^ However, this typically requires specialized high‐surface‐area materials like carbon nanotubes, which complicates synthesis and limits scalability.

Molecular‐level structural modification offers a promising alternative. Introducing peripheral groups such as methyl (─CH_3_) or amino (─NH_2_) can improve conductivity, but since these modifications do not alter the metal center's coordination environment, the performance gains are limited.^[^ ^18^, ^27^, ^28^, ^29^ ^]^ Alternatively, constructing 2D polyphthalocyanine networks can enhance conductivity, though this approach often suffers from structural disorder, insolubility, and difficulties in characterization, posing significant challenges for practical application.^[^ ^19^, ^30^ ^]^

To overcome the aforementioned challenges, we propose a novel molecular design strategy by introducing pyridinic‐N atoms at the β‐positions of CoPc macrocyclic backbone. Through a concise synthesis, we developed cobalt tetra‐aza‐phthalocyanine (CoTAP), a new Pc derivative that exhibits a ≈27‐fold enhancement in conductivity compared to pristine CoPc. When supported on commercial Ketjenblack (KB) and assembled into gas diffusion electrodes (GDEs), the resulting CoTAP GDE delivers ≈100% CO selectivity over a broad potential window (−0.25 to −1.45 V vs RHE), with an ultralow onset overpotential of just 140 mV (−0.25 V vs RHE), significantly outperforming CoPc (−0.57 V vs RHE). Such improvement is attributed to both enhanced conductivity and modulation of the cobalt center's electronic environment by the incorporated pyridinic‐N. Density functional theory (DFT) calculations reveal that CoTAP offers more optimal binding energies for key intermediates (^*^COOH and ^*^CO), placing it closer to the peak activity of the volcano model and confirming its superior intrinsic activity. As a result, CoTAP achieves a record‐breaking CO current density of −1084 mA cm^−2^ and an exceptional mass activity (MA) of 24,636.4 A g^−1^, along with excellent long‐term stability for 112 h at −150 mA cm^−2^, surpassing all reported Pc‐based catalysts. Given the comparable surface areas at optimal loadings, CoTAP also shows a significantly higher turnover frequency (TOF) of 73.4 s^−1^ compared to 12.1 s^−1^ for CoPc, experimentally validating the enhanced site‐specific intrinsic activity in CoTAP. These results underscore the effectiveness of pyridinic‐N incorporation in boosting both the conductivity and intrinsic catalytic performance of Pc‐based electrocatalysts for industrial ECR.

## 2 Results and Discussion

### 2.1 Strategical Synthesis and Structural Characterization of CoTAP

**Figure 1:**
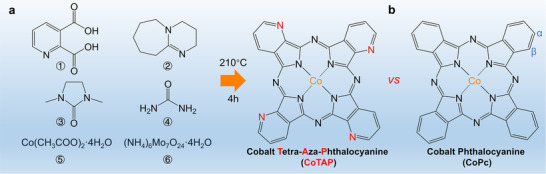
Synthesis and structure of cobalt tetra‐aza‐phthalocyanine (CoTAP). a) Schematic illustration of the rapid synthesis of CoTAP from basic starting materials. b) Structural comparison between CoTAP and pristine CoPc.

**Figure 2:**
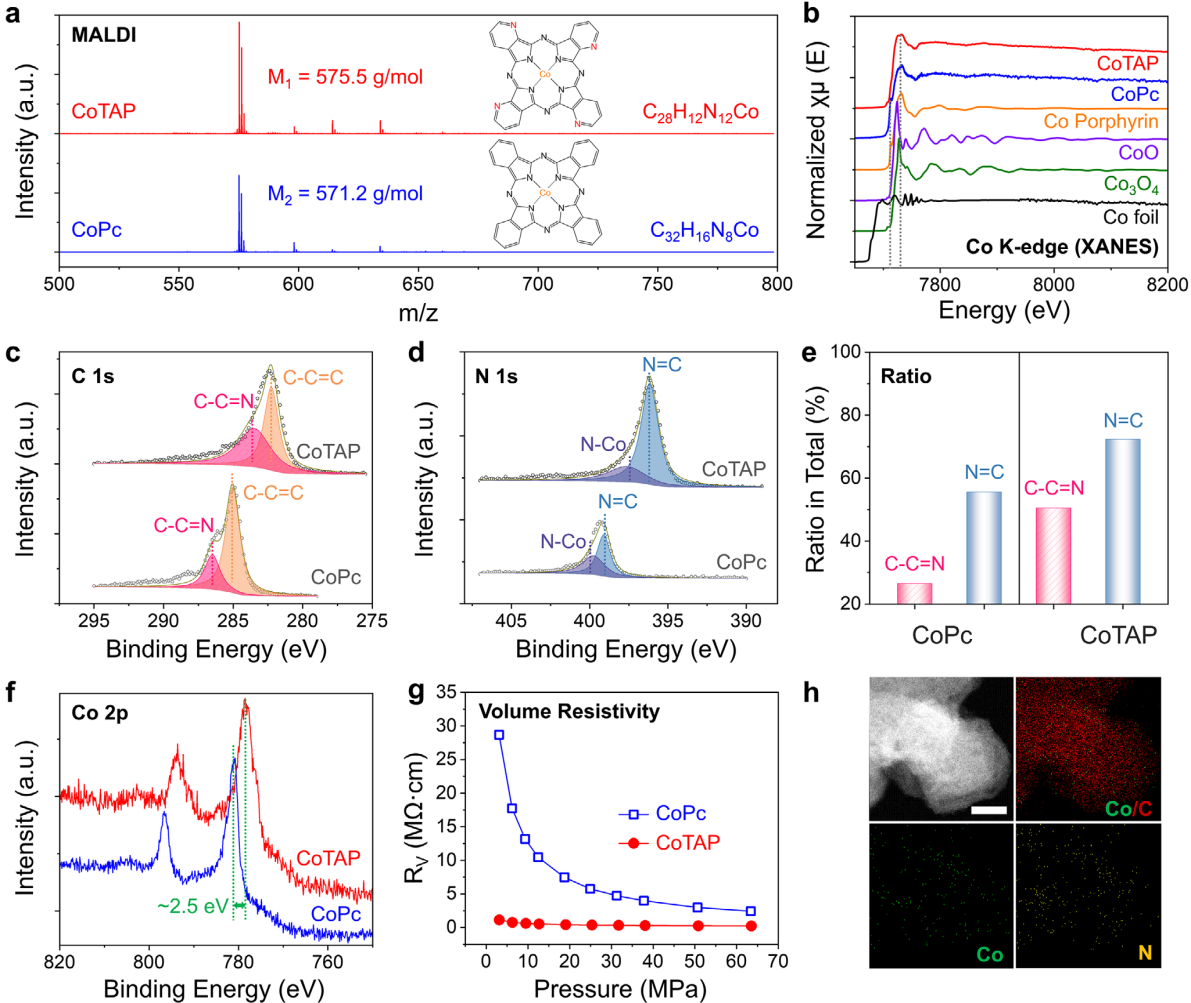
Structural characterization and property comparison between CoTAP and pristine CoPc. a) MALDI‐TOF‐MS analysis confirming the molecular weights of CoTAP and pristine CoPc, consistent with their corresponding molecular structures. b) The Co K‐edge XANES spectra of CoTAP and reference (CoPc, Co‐porphyrin, CoO, Co_3_O_4_, and Co foil). c, d) The XPS spectra of CoTAP and CoPc in the C 1s (c) and N 1s (d) regions. e) Relative peak area of C═N bonds in total N and C spectra: CoPc (left) vs. CoTAP (right). f) Binding energy shift in the Co 2p orbitals between CoTAP and CoPc. g) Comparison of volume resistivity as a function of applied pressure for CoTAP and CoPc. h) Elemental mapping of Co, N, and C, scale bar: 10 nm.

Inspired by our previous work on Pcs synthesis,^[^ ^20^, ^31^, ^32^ ^]^ we strategically introduce 1,8‐diazabicyclo [5.4.0] undec‐7‐ene, an efficient organic precursor containing pyridinic‐N, into the reaction. This approach enables the successful incorporation of pyridinic‐N at the β‐positions of the CoPc macrocyclic backbone, yielding the target product, CoTAP (**Figure** **1**). The detailed synthetic procedure is provided in the Experimental Section of the Supporting Information (SI). Matrix‐assisted laser desorption/ionization time‐of‐flight mass spectrometry (MALDI‐TOF‐MS) analysis confirms that the synthesized CoTAP possesses a molecular weight of 575.5 g mol^−1^, which is in excellent agreement with the theoretical value of 575.4 g mol^−1^ based on its molecular formula, C_28_H_12_N_12_Co (**Figure** **2**a). In comparison, pristine CoPc displays a measured molecular weight of 571.2 g mol^−1^, consistent with its calculated value derived from the molecular formula C_32_H_16_N_8_Co. These results verify the successful substitution of four β‐position carbon atoms with nitrogen, resulting in the formation of the desired CoTAP structure.

X‐ray diffraction (XRD) analysis reveals that pristine CoPc exhibits a typical β‐CoPc crystalline form (PDF No. 14–948), whereas the diffraction peaks of CoTAP differ markedly in both position and intensity (Figure S1, Supporting Information). These differences suggest that the incorporation of pyridinic‐N atoms, with their additional lone‐pair electrons, modifies the intermolecular *π*–*π* stacking and alters the electron density distribution. Consequently, the crystal structure becomes distorted and the molecular packing changes, leading to peak broadening and intensity variation in the XRD pattern.

In addition, X‐ray absorption near edge structure (XANES) spectra further reveal changes in the coordination environment at the Co centers induced by pyridinic‐N incorporation (Figure 2b). Consistent with our previous findings, pristine CoPc exhibits a peak shape and position similar to that of cobalt porphyrin but clearly distinct from cobalt oxides, confirming its well‐defined Co‐N_4_ structure. CoTAP shows an identical absorption peak at 7730 eV, indicating that the Co‐N_4_ coordination site is preserved during synthesis. Interestingly, compared to pristine CoPc, CoTAP exhibits a noticeable shift toward higher energy at the absorption edge near 7712 eV, indicating that the incorporation of pyridinic‐N has altered the electronic environment of the Co center and enhanced its electron affinity.^[^ ^33^ ^]^

X‐ray photoelectron spectroscopy (XPS) analysis further confirms the binding energy shifts induced by structural modifications. To ensure transparency, the raw, unprocessed XPS data, which are exactly as obtained from the instrument, are presented in Figures S2 and S3 (Supporting Information). Additional XPS measurements were performed on Pc‐powder/Si systems, prepared by directly depositing CoPc and CoTAP powders onto single‐crystal Si substrates. Survey spectra reveal that both CoTAP and pristine CoPc contain the expected elements C, N, and Co (Figures S2a and S3a, Supporting Information). Notably, CoTAP exhibits a substantially higher nitrogen content than CoPc, as indicated by the relative peak intensities, consistent with their molecular structures. Background‐corrected UV–vis spectroscopy spectra further reveal that, compared with CoPc, CoTAP exhibits a distinct red shift in the B band (≈370 nm) and a blue shift in the Q band (550–700 nm) (Figure S4, Supporting Information). More importantly, CoTAP displays a unique absorption feature in the 720–900 nm region, which may arise from electronic density redistribution induced by the incorporation of pyridinic‐N or from charge‐transfer (CT) transitions. Consistent differences are also observed in the Fourier‐transform infrared (FTIR) spectroscopy spectra, where a new peak at ≈1600 cm^−1^, attributable to pyridinic‐N incorporation, is present in the CoTAP pattern (Figure S5, Supporting Information). Together, these results provide strong evidence that pyridinic‐N has been successfully incorporated into the CoPc backbone, in agreement with the preceding analytical findings.

After curve fitting, the C 1s spectra were resolved into two components corresponding to C─C═C and C─C═N species (Figure 2c), while the N 1s spectra were deconvoluted into N═C and N─Co species (Figure 2d).^[^ ^34^, ^35^ ^]^ Upon normalization, CoTAP displays markedly larger peak areas for both C─C═N and N═C compared to CoPc (Figure 2e), reflecting the increased nitrogen content and verifying the successful incorporation of pyridinic‐N atoms into the macrocyclic framework, in agreement with MALDI‐TOF, XANES, and XRD results. Importantly, CoTAP shows a distinct ≈2.5 eV shift in the binding energies of the C 1s, N 1s, and Co 2p regions relative to CoPc (Figure 2f; Figures S2b–d and S3b–d, Supporting Information), while the Si 2p peaks remain unchanged (Figures S2e and S3e, Supporting Information), indicating enhanced electron density arising from pyridinic‐N incorporation. This electron enrichment contributes to improved conductivity. Furthermore, as shown in our previous study,^[^ ^23^ ^]^ electron donation to the Co center not only modulates its intrinsic activity but also optimizes the adsorption energies of key intermediates, thereby enhancing catalytic performance.

To verify the enhanced conductivity, we measured the volume resistivity (*R*_V_) of both materials (Figure 2g). Without applied pressure, CoPc exhibits an *R*_V_ of 28.7 MΩ cm, whereas CoTAP shows a significantly lower value of just 1.1 MΩ cm, which is only 3.8% of that of pristine CoPc. Upon applying external pressure, the resistivity of CoPc decreases markedly, while CoTAP remains nearly unchanged. These results indicate that CoTAP possesses inherently superior electronic conductivity, supported by stable charge transport pathways and favorable *π*–*π* stacking or charge migration channels. Based on its superior electronic properties, CoTAP molecules are dispersed onto commercial Ketjenblack (KB) carbon black to construct a CoTAP/KB hybrid with single‐molecule‐level dispersion. Elemental mapping clearly demonstrates the uniform distribution of Co across the C matrix (Figure 2h), indicating that this strategy effectively establishes a high‐performance catalytic system with well‐distributed active sites and continuous electron transport pathways.

2D small‐angle X‐ray scattering (SAXS) further reveals distinct nanoscale structures among the samples (Figure S6, Supporting Information). Pristine CoPc and CoTAP powders show sharp ring‐like patterns indicative of strong crystallinity, but with different semi‐ring diameters, reflecting distinct crystal structures. Blank KB displays a diffuse‐halo pattern characteristic of amorphous structures. The CoPc/KB hybrid resembles KB, indicating well‐dispersed, non‐crystalline CoPc. In contrast, CoTAP/KB retains CoTAP diffraction peaks, likely due to pyridinic‐N–driven intermolecular interactions forming “polyphthalocyanine”‐type assemblies. SAXS curves (Figure S7, Supporting Information) confirm these observations: KB shows two peaks in the Q range of 10–13 nm^−1^, while CoPc/KB and CoTAP/KB exhibit shifted peaks, suggesting preferential growth along KB surfaces. Additional peaks in CoTAP/KB match crystalline CoTAP, further indicating molecule‐molecule interactions enhanced by its higher conductivity.

### 2.2 Electrocatalytic CO_2_ Reduction Performance of CoTAP

**Figure 3:**
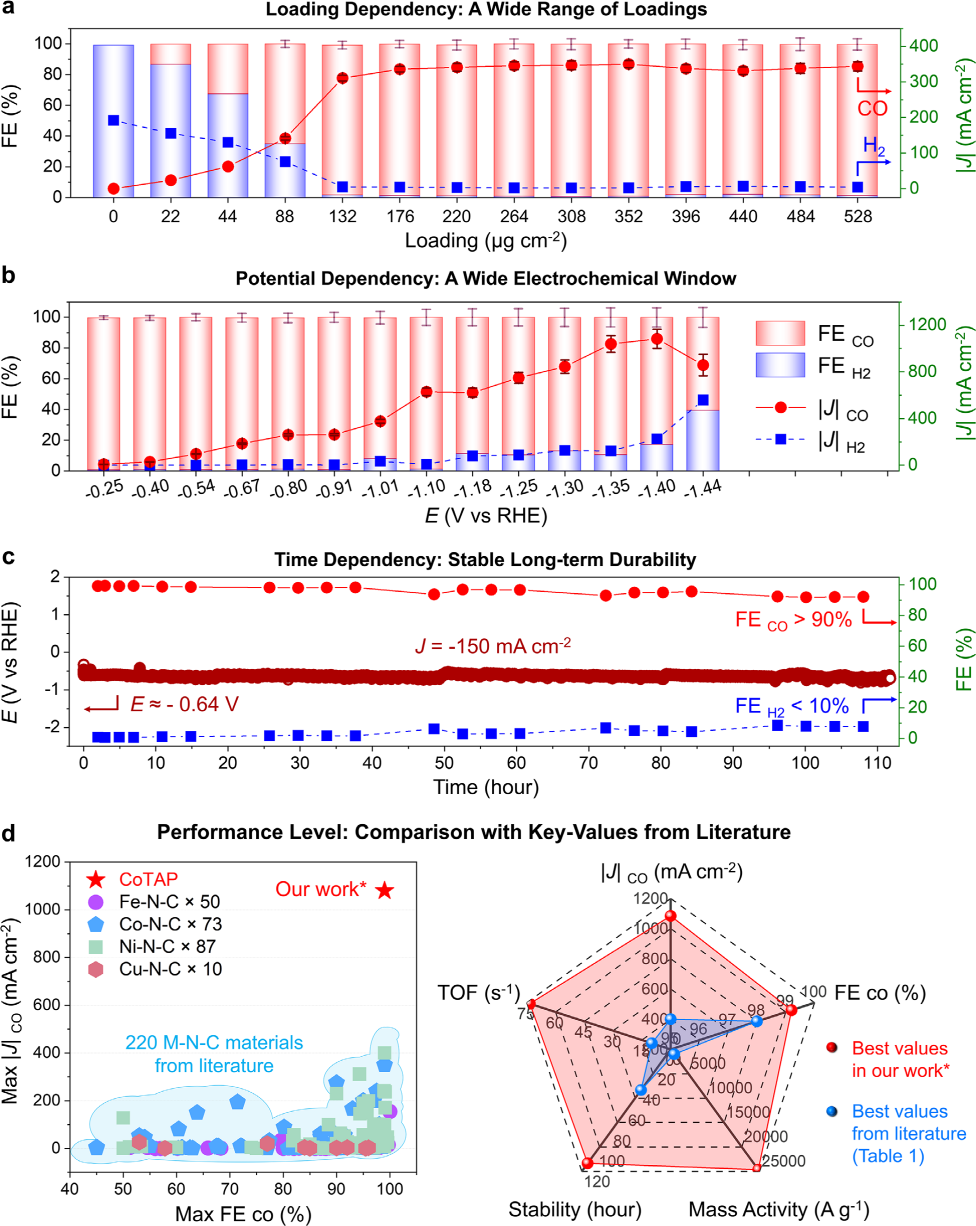
Electrocatalytic performance of CoTAP GDEs for ECR. a) Faradic efficiency (FE, left axis) and absolute current density (|*J*|, right axis) for CO (red) and H_2_ (blue) for electrodes at different loadings, at a potential (*E*) of −0.91V (vs. RHE). b) FE (left axis) and |*J*| (right axis) as a function of *E* (vs. RHE) for electrodes at a loading of 220 µg cm^−2^. c) Long‐term stability of the CoTAP GDE at a constant *J* of −150 mA cm^−2^, showing variation in *E* (left axis) and FE (right axis). d) Comparison of maximum FE and |*J*| for CO in our work with other literature values (left), and radar plots showing best metrics from our work (red) vs the other literature (blue) (right).^[^ ^35^, ^36^, ^37^, ^38^, ^39^, ^40^, ^41^, ^42^, ^43^ ^]^

Based on our previous protocol, 300 mg of CoTAP/KB hybrid catalyst (20 wt%) is dispersed in 90 mL of solution and spray‐coated onto MFK‐A carbon paper to construct a gas diffusion electrode (GDE). The actual catalyst loadings are precisely controlled by varying the number of spray passes. After cutting and masking, the fabricated GDEs are employed as the cathode in a flow‐cell electrolyzer for electrochemical CO_2_ reduction (ECR) testing (Scheme S1, Supporting Information). We systematically investigate the impact of catalyst loading on ECR performance. As shown in **Figure** **3**a, even a single spray pass with an ultra‐low loading of ≈22 µg cm^−2^ (4.4 µg cm^− 2^ of CoTAP) yields measurable CO production, highlighting CoTAP's high intrinsic activity. Increasing spray passes raises the loading and number of active sites, thereby enhancing CO output. At a loading of ≈ 132 µg cm^−2^ (six passes), the CO Faradaic efficiency (FE_CO_) exceeds 98%, making it nearly the exclusive product. Beyond this, total current densities (*J*_total_) continue to rise until leveling off at ten passes (≈220 µg cm^−2^), indicating saturation of active sites at the gas‐liquid‐solid boundary, with further catalyst addition becoming redundant.

Further potential‐dependent tests are conducted using the ten‐pass GDEs (≈220 µg cm^−2^ loading), and the corresponding *J*–*t* curves are shown in Figure S8 (Supporting Information). As shown in Figure 3b, CoTAP exhibits an ultralow onset overpotential of just 140 mV (−0.25 V vs RHE) with ≈100% CO selectivity, significantly outperforming pristine CoPc (−0.57 V vs RHE) and highlighting its higher intrinsic activity. As the potentials become more negative, the CO production rates increase sharply, reaching a peak CO current density (*J*_CO_) of −1084 mA cm^−2^ at −1.40 V vs RHE—a record among Pc‐based catalysts. In comparison, CoPc delivers only −595 mA cm^−2^ (Figure S9, Supporting Information). According to the relation *J*_partial_ = *J*_total_ × FE, this high *J*_CO_ results from both a large total current and high selectivity. The high *J*_total_ reflects an abundance of accessible active sites and an optimized triple‐phase interface, while the high FE arises from superior molecular‐level catalytic activity. Together, these features account for the outstanding performance of CoTAP.

In addition to its high current density, Figure 3c shows the long‐term stability of the CoTAP GDE during continuous electrolysis at −150 mA cm^−2^, with the applied potential remaining nearly constant at −0.64 V vs RHE over 110 h, demonstrating excellent durability. Minor fluctuations are attributed to operational factors such as slight GDE flooding rather than catalyst degradation. Post‐reaction XPS analysis (Figure S10, Supporting Information) reveals no significant changes in the Co 2p spectra before and after electrolysis, confirming that the active CoTAP sites are well preserved during CO_2_ reduction.

The above results provide compelling evidence that our strategically engineered pyridinic‐N incorporated CoPc (CoTAP) exhibits markedly enhanced performance compared to its unmodified parent compound (pristine CoPc). To further contextualize its catalytic performance, **Table** **1** summarizes key parameters of representative Pc‐based electrocatalysts reported in recent years, as extracted from the DigCat Database (https://www.digcat.org/), the largest curated electrocatalysis database to date. For ease of comparison, maximum *J*_CO_ and FE_CO_ values are highlighted in Figure 3d (left). These data clearly show that CoTAP outperforms all previously reported Pc‐based catalysts. Importantly, previous studies suggest that practical industrial applications require a *J*_partial_ above 500 mA cm^−2^ and an FE greater than 98%. Only CoTAP satisfies both criteria, while other reported systems fall short of the −500 mA cm^−2^ benchmark. Additional comparisons of key metrics such as TOF and MA are also summarized in Table 1 and visualized in the radar plots (Figure 3d, right). Notably, CoTAP achieved a record‐high mass activity of 24,636.4 A g^−1^, which is ≈24 times higher than the previously reported maximum of 1000 A g^−1^,^[^ ^36^ ^],^ demonstrating exceptional catalytic efficiency per unit mass. In addition, CoTAP delivered a TOF of 73.4 s^−1^, significantly exceeding the prior best of 9.8 s^−1^,^[^ ^34^ ^]^ indicating highly efficient utilization of each active site. In addition, we collected key performance metrics of state‐of‐the‐art non‐phthalocyanine (non‐Pc) catalysts for CO electrosynthesis reported in recent years. As shown in Table S3 (Supporting Information), our CoTAP catalyst demonstrates superior performance compared with these non‐Pc catalysts. Taken together, these results underscore the strong potential of CoTAP for industrial‐scale applications.

**Table 1:** Comparison of the key values in this work and selected literature.

Catalyst	pH	FE_CO_ [%]	*J*_CO_ [mA cm^−2^]	TOF [s^−1^]	MA [A g^−1^]	Stability @ *J* [h @ mA cm^−2^]	Refs.
CoTAP/KB	14.0	>98.0	−1084	73.4	24636.4	112 @ −150	This work^*^
CoPc/KB	14.0	>98.0	−595	12.1	6537.4	100 @ −100	Our work^[^ ^22^ ^]^
SCT‐CoPc Crystals	14.0	>98.0	−1036	13.8	5180.0	>100 @ −150	Our work^[^ ^23^ ^]^
CoPc/CNT‐MDE	6.8	98.0	−15	4.1	37.5	10 @ −10^a)^	[43]
CoPc/CNT‐MDE	2.0	73.0	−38	–	47.5	27 @ −90^a)^	[44]
CoPc/CB‐MDE	7.8	98.0	−100	–	–	10 @ −3^a)^	[41]
CoPc/CNT‐MDE	6.8	97.0	−200	–	–	38 @ −250	[39]
CoPc/CNT‐ODA	7.3	97.7	−350	–	350	12 @ −150^a)^	[40]
CoPc‐TBG/CNT	14.0	96.0	−112	2.7	560	3 @ −150	[42]
CoPc‐ EtO_8_/CNP	7.8	95.0	−340	–	–	24 @ −150	[35]
CoPc‐OCH_3_/CNT	7.3	97.0	−280	9.8	–	10 @ −150	[34]
CoPPc/CNT	7.3	90.0	−19	1.25	19	24 @ −15	[45]
NiPc/CNT‐MDE	7.3	>98.0	−400	–	1000	40 @ −150	[36]
NiPc/NHCSs	7.3	98.6	−25	–	25	32 @ −100	[46]
NiPc(OH)_6_(DCNFO)/CNT	14.0	>98.0	−380	3.3	–	40 @ −150	[38]
NiPc‐OMe	2.0	>98.0	−400	–	400	12 @ −100	[37]

^a)^ The experiments are conducted under constant voltage, so the *J* values are estimated from the corresponding *J*–*t* curves.

### 2.3 Mechanistic Insights into the Enhanced Activity of CoTAP

To preliminarily investigate the electrocatalytic mechanism, we compared the electrochemical performance under CO_2_ and Ar atmospheres (Figure S11, Supporting Information). First, the LSV curves of both CoTAP/KB and CoPc/KB exhibit higher current densities under CO_2_ than under Ar, reflecting differences in reaction rates. Notably, CoTAP/KB shows higher current densities than CoPc/KB, indicating enhanced catalytic activity. Under a CO_2_ atmosphere, over 98% CO selectivity was observed, whereas under Ar, only H_2_ was detected (Figure S12, Supporting Information). All other conditions were kept constant, with the gas atmosphere as the sole variable, confirming that the CO originates from the supplied CO_2_.

Additionally, a smaller Tafel slope was observed for CoTAP compared with CoPc, indicating faster reaction kinetics in the electrochemical CO_2_ reduction process (Figure S13, Supporting Information). A lower Tafel slope generally suggests that the rate‐determining step involves a reduced activation barrier, facilitating more efficient electron and proton transfer. Electrochemical impedance spectroscopy (EIS) measurements (Figure S14, Supporting Information) show that CoTAP‐based GDEs possess markedly lower charge‐transfer resistance than their CoPc counterparts. Taken together, these results provide strong evidence that the conductivity advantage from pyridinic‐N incorporation is retained in the electrode system.

**Figure 4:**
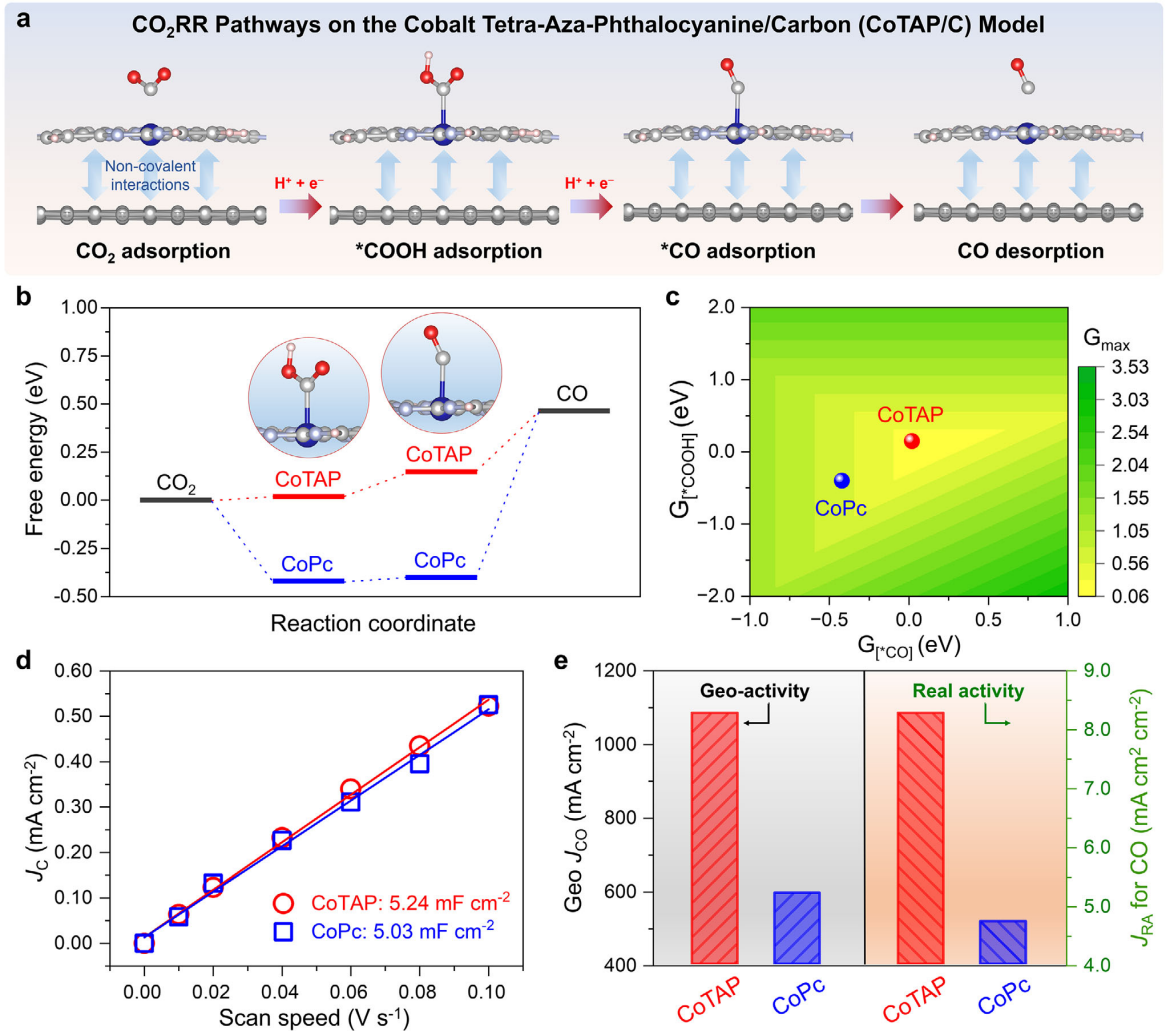
Mechanistic insights into the enhanced ECR performance of CoTAP compared with conventional CoPc. a) Proposed reaction pathway from CO_2_ to CO on the CoTAP supported on carbon (CoTAP/C) model. b) DFT‐calculated adsorption free energies (G_[*COOH]_ and G_[*CO]_) for CoTAP/C and CoPc/C structures. c) Volcano model of ECR as the function of G_[*COOH]_ and G_[*CO]_, illustrating the intrinsic activity enhancement of CoTAP. d) Double‐layer capacitance (C_dl_) of CoTAP and CoPc GDEs. e) Maximum CO production current density based on geometric area (left) and real intrinsic activity normalized by electrochemical surface area (right), demonstrating the superior per‐site activity of CoTAP over CoPc.

Beyond this enhanced conductivity, we propose that pyridinic‐N also strengthens the intrinsic catalytic activity of the Co center. To test this, DFT calculations were performed on CoTAP/C and CoPc/C models to evaluate the Gibbs free energies (ΔG) of key CO_2_ reduction intermediates, ^*^COOH and ^*^CO. As illustrated in **Figure** **4**a, four key stages are modeled: CO_2_ adsorption, ^*^COOH adsorption, ^*^CO adsorption, and CO desorption. The calculated ΔG values reveal that CoPc binds ^*^COOH and ^*^CO with energies of −0.42 and −0.40 eV, respectively, whereas CoTAP shows more favorable adsorption energies of 0.02 and 0.14 eV (Figure 4b). These values indicate weaker binding of intermediates on CoTAP, which enables easier desorption and faster catalytic turnover. Furthermore, the volcano model positions CoTAP closer to the theoretical activity apex, further corroborating its superior intrinsic catalytic performance (Figure 4c).

We further extend our study by increasing the number of pyridinic N atoms to eight within the CoPc backbone, generating a cobalt octa‐aza‐phthalocyanine (CoOAP) model. The binding energies of key reaction intermediates were then calculated on this CoOAP structure. The results (Figure S15, Supporting Information) indicate that, although CoOAP contains a higher pyridinic N content, it exhibits less favorable DFT‐calculated adsorption free energies compared with CoTAP/C. These findings suggest that CoTAP achieves an optimal balance in adsorption energies, which accounts for its superior catalytic performance, whereas further increasing the pyridinic N content, as in CoOAP, appears to be excessive and results in suboptimal activity.

To further clarify the enhancement in intrinsic activity, cyclic voltammetry (CV) measurements are conducted at various scan rates within the non‐faradaic potential region to determine the double‐layer capacitance (*C*_dl_), thereby enabling the estimation of the electrochemically active surface area (ECSA) for both electrodes (Figures S16 and S17, Supporting Information).^[^ ^47^, ^48^ ^]^ The extracted *C*_dl_ values for CoTAP and CoPc are 5.24 and 5.03 mF cm^−2^, corresponding to ECSA values of 175 and 173 cm^2^ cm^−2^, respectively, indicating nearly identical active site densities at equal loading (Figure 4d). Under their respective optimal conditions, CoTAP achieves a maximum |*J*|_CO_ of 1084 mA cm^−2^ (geometric area), compared to 595 mA cm^−2^ for that of CoPc (geometric area). When normalized by ECSA, CoTAP exhibits an intrinsic current density of 8.7 mA unit^−1^, nearly double that of CoPc (4.5 mA unit^−1^) (Figure 4e). In addition, CoTAP delivers a turnover frequency of 73.4 s^−1^, markedly outperforming that of CoPc (12.1 s^−1^). These findings collectively underscore that the superior electrocatalytic performance of CoTAP originates primarily from its enhanced intrinsic activity at the molecular active sites.

## 3 Conclusion

In this study, we design and synthesize a novel CoPc‐derivative (CoTAP) by introducing four pyridinic‐N atoms at the β‐positions of the macrocyclic backbone. This molecular modification significantly enhances electrical conductivity, reducing the bulk resistivity to just 3.8% of that of pristine CoPc, while simultaneously modulating the electronic environment at the cobalt centers. When employed as the ECR cathode, our CoTAP electrode exhibits ≈100% CO selectivity across a broad potential window (−0.25 to −1.45 V vs. RHE), with an ultralow onset overpotential of 140 mV (−0.25 V vs RHE), demonstrating substantially higher activity than CoPc. Furthermore, it also delivers a record‐high CO current density of −1084 mA cm^−2^, an ultrahigh mass activity of 24,636.4 A g^−1^, and outstanding long‐term operational stability over 112 h at −150 mA cm^−2^, surpassing all reported Pc‐based catalysts to date. DFT calculations reveal that the incorporation of pyridinic‐N optimizes the adsorption free energies of key reaction intermediates (^*^COOH and ^*^CO), shifting CoTAP closer to the theoretical activity apex for CO production. Given the comparable electrochemical surface areas at optimal loadings, CoTAP also exhibits a significantly higher turnover frequency of 73.4 s^−1^ compared to 12.1 s^−1^ for CoPc, validating its superior intrinsic activity on a per‐site basis. Overall, this work highlights pyridinic‐N incorporation as a powerful design strategy to simultaneously enhance conductivity and catalytic performance in Pc‐based materials for efficient CO_2_ electroreduction.

## Conflict of Interest

The authors declare no conflict of interest.

## Author Contributions

T.L., X.H., and D.Z. contributed equally to this work. T.L. conducted conceptualization, investigation, methodology, resources, formal analysis, and writing of the original draft. X.H. conducted formal analysis. D.Z. handled conceptualization and methodology. Y.H., K.I., Y.M., J.Y., and S.O. participated in methodology, resources, and formal analysis. H.L. and H.Y. led conceptualization, resources, writing—review & editing, and supervision. T.L., H.L., and H.Y. coordinated collaboration among all co‐authors and finalized the draft. All authors contributed to the revisions.

## Supporting information



Supporting Information

## Data Availability

The data that support the findings of this study are available in the Supporting Information of this article.
