# Supplementary material for: Ampere‐Level Electrosynthesis of CO via Well‐Defined Pyridinic‐N Incorporated Cobalt Phthalocyanine
Source: Small. 2025 Sep 30;21(47):e07824. doi: 10.1002/smll.202507824 (PMC12658917; doi:10.1002/smll.202507824)
Supplement: Supplementary file 1 — Supporting Information [file SMLL-21-e07824-s001.pdf]

NANO · MICRO
small

Supporting Information

for *Small*, DOI 10.1002/smll.202507824

Ampere-Level Electrosynthesis of CO via Well-Defined Pyridinic-N Incorporated Cobalt Phthalocyanine

Tengyi Liu, Xiaofan Hou, Di Zhang, Yutaro Hirai, Kosuke Ishibashi, Yasutaka Matsuo, Junya Yoshida, Shimpei Ono, Hao Li* and Hiroshi Yabu**

The Supporting Information for

Ampere-Level Electrosynthesis of CO via Well-Defined Pyridinic-N Incorporated Cobalt Phthalocyanine

Tengyi Liu,[†,] Xiaofan Hou,[†] Di Zhang,[†] Yutaro Hirai, Kosuke Ishibashi, Yasutaka Matsuo, Junya Yoshida, Shimpei Ono, Hao Li,* and Hiroshi Yabu**

*** Corresponding Authors:**

T. Liu, Assistant Professor, WPI-AIMR, Tohoku University, Sendai, Japan; Tel/Fax: +81-022-217-6341; Email: liu.tengyi.e1@tohoku.ac.jp; Orcid: 0000-0003-3581-1842.

H. Li, Professor/Principal Investigator, WPI-AIMR, Tohoku University, Sendai, Japan; Tel/Fax: +81-022-217-6371; Email: li.hao.b8@tohoku.ac.jp; Orcid: 0000-0002-7577-1366.

H. Yabu, Professor/Principal Investigator, WPI-AIMR, Tohoku University, Sendai, Japan; Tel/Fax: +81-022-217-5996; Email: hiroshi.yabu.d5@tohoku.ac.jp; Orcid: 0000-0002-1943-6790.

[†] These authors contributed equally to this work.

Contents

S-1. Experiment Section 3
S-1.1. Materials and Regents 3
S-1.2 Equipment and Devices 3
S-1.3. Synthesis and Preparation 4
S-1.3-1. Synthesis of CoTAP 4
S-1.3-2. Preparation of Carbon-Supported CoTAP Hybrid 4
S-1.3-3. Preparation of CoTAP/KB Electrodes 5
S-1.4. Electrochemical CO_2 Reduction (ECR) Test 5
S-1.5. Qualitative and Quantitative Analysis of ECR Products 6
S-1.6. Computational methods 7
Scheme S1. Laboratory electrochemical CO_2 reduction (ECR) system 9
Table S1. Chemical reactions and numbers of electrons transferred 10
Figure S1. XRD patterns of CoTAP, CoPc and standard references. 11
Figure S2. Unprocessed XPS spectra of CoPc powder on a Si substrate 12
Figure S3. Unprocessed XPS spectra of CoTAP powder on a Si substrate 13
Figure S4. UV-vis-NIR spectra 14
Figure S5. Typical FTIR patterns 15
Figure S6. Typical 2D SAXS patterns (Q-zone) 16
Figure S7. Typical 1D-integrated SAXS patterns 17
Figure S8. Time-dependent total current density curves 18
Figure S9. Electrocatalytic performance of CoPc/KB GDE 19
Table S2. Comparation of the key-values in this work and selected literature 20
Table S3. Comparation of the key-values from non-Pc-based catalysts 21
Figure S10. Co 2p spectra of the CoTAP/KB electrode 22
Figure S11. LSV curves of CoTAP/KB and CoPc/KB GDEs 23
Figure S12. FEs of CoTAP/KB GDEs under CO_2 and Ar atmospheres 24
Figure S13. Tafel slopes of CoTAP/KB and CoPc/KB electrodes 25
Figure S14. The in situ electrochemical impedance spectroscopy (EIS) measurements 26
Figure S15. Comparison of free energies for CoTAP and CoOAP 27
Figure S16. ECSA measurement of CoTAP/KB electrodes. 29
Figure S17. ECSA measurement of CoPc/KB electrodes. 29

S-1. Experiment Section

S-1.1. Materials and Regents:

CoTAP and CoPc were synthesized or supplied by Azul Energy Corporation, each with a purity higher than 99.9%. Ketjen-Black carbon black (EC-600JD) was sourced from Fuel Cell Store. 2-Propanol (IPA) and dimethyl sulfoxide (DMSO) were obtained from Fuji-Wako, each with a purity above 99.5%. Nafion dispersion solution (20 wt%) was purchased from Sigma-Aldrich. The carbon paper gas diffusion layers used for electrochemical CO_2 reduction tests were acquired from Mitsubishi Chemical Corporation (PYROFIL-GDL MFK-A, 0.21 mm), while those (ABOUND-GDL) for long-term durability tests were supplied by Abound Energy Corporation. All gases, including carbon dioxide (CO_2), carbon monoxide (CO), methane (CH_4), ethylene (C_2H_4), and hydrogen (H_2), were purchased from Taiyo Nippon Sanso Company, each with a purity over 99.9%. Ultrapure water (18.2 MΩ) was obtained through a purification system. All reagents were used as received without further purification.

S-1.2 Equipment and Devices:

The following equipment was used in this study. A ball-mill (FRITSCH, Planetary Mono Mill Pulverisette 6) ground materials into fine powder, while a sim-coat system (Sono-Tek) applied uniform catalyst films onto substrates. Gas chromatography with flame ionization detection (GC-FID, Shimadzu GC-2014) analyzed gaseous products, and an electrochemical workstation (Princeton Applied Research, Versa-STAT 4) conducted electrochemical measurements, including CO_2 reduction tests. An analytical balance (A&D, BM-20) ensured precise mass measurements, and an oven (Espec, ST-120) was used for controlled drying. An ultrasonic machine (Asone, ASU-2M) dispersed inks, and gas flow meters (FCON, C2005-CO_2-003) monitored gas flows. Ultrapure water was generated using an ELGA (LA-758 Purelab system) purification system. X-ray photoelectron spectroscopy (XPS, Ulvac-PHI 5000 Versa Probe II) analyzed surface composition, X-ray diffraction (XRD, RIGAKU Smart-Lab) determined crystalline structures, and X-ray absorption fine structure (XAFS, SPring-8 & NanoTerasu) spectroscopy provided local electronic insights. The raw data were analyzed using

the ATHENA software packages. Fourier transformations of the k^3-weighted EXAFS spectra were conducted for all samples over a k-space range of 3–10 $Å^{-1}$. For the resulting data, analyzing the R-space range from 0 to 6 Å (or up to 10 Å if needed) was deemed sufficient in this work. A scanning electron microscope (SEM, JEOL JSM-7800F) and field emission electron probe microanalysis (FE-EPMA, JEOL JXA-8530F) were used for imaging and elemental analysis.

S-1.3. Synthesis and Preparation:

S-1.3-1. Synthesis of CoTAP: As shown in **Figureure 1**, the chemical structures and corresponding compound numbers (1 to 6) are illustrated in sequential order. For the synthesis, 4.50 g of quinolinic acid (26.9 mmol), 32.8 mg of 1,8-diazabicyclo [5.4.0] undec-7-ene (2.15 mmol), and 30 mL of 1,3-dimethyl-2-imidazolidinone, 12.94 g of urea (215.4 mmol), 1.677 g of cobalt (II) acetate tetrahydrate (6.7 mmol), and 31 mg of ammonium molybdate tetrahydrate (0.0269 mmol), were introduced into a 100 mL three-necked round-bottom flask. The mixture was stirred and heated at 210 °C for 4 hours under a continuous flow of nitrogen. Upon completion of the reaction, the mixture was cooled to 80 °C and filtered under vacuum. The resulting solid was sequentially washed with acetone, methanol, and water, and then dried under reduced pressure at 60 °C to afford the desired product. The final yield was 52.7%.

S-1.3-2. Preparation of Carbon-Supported CoTAP Hybrid: In this work, CoTAP/KB catalysts were prepared using a method further refined from our previous report [1]. For instance, a 20 wt% CoTAP/KB catalyst was synthesized by first dissolving 200 mg of CoTAP in 150 mL of DMSO in a round-bottom flask, followed by the addition of 800 mg of Ketjen Black (KB), maintaining a total solid mass of 1000 mg. This fixed solid-to-solvent ratio was kept constant across all samples. The resulting mixture was sonicated in an ice-water bath for 30 minutes to promote the adsorption of CoTAP onto the KB surface. Sonication was carried out using a probe sonicator set to an amplitude of 1 and a 30% pulse cycle, as higher amplitudes could result in the dissolution of CoTAP rather than its adsorption. After adsorption, the mixture was filtered, and the solid was washed three times with methanol, then dried at a moderate temperature (e.g.,

35 °C) in a vacuum oven for 24 hours to preserve catalytic activity. The dried product was ground using a mortar, weighed, and transferred to a ball-mill autoclave. The ball-milling solvent consisted of DMSO (9 mL), IPA (9 mL), H_2O (4.5 mL) and Nafion (20 wt%, 0.1 mL per 50 mg catalyst). Ball milling was conducted at 400 rpm for 5 minutes, followed by reverse rotation for another 5 minutes, repeated for a total of 30 minutes to reduce carbon agglomeration. After milling, the ball-mill autoclave was rinsed with a solvent mixture containing DMSO (9 mL), IPA (45 mL), and H_2O (13.5 mL), bringing the total solvent volume to 90 mL. The resulting mixture was then ultrasonically dispersed for at least 30 minutes to obtain a uniform catalyst ink.

S-1.3-3. Preparation of CoTAP/KB Electrodes: The resulting ink was spray-coated onto carbon paper to fabricate the CoTAP/KB hybrid electrode. Different catalyst loadings were achieved by adjusting the ink concentration and the number of spray passes. A slim-coating system (Flex-Coat Max, Sono-Tek) was used for spray-coating. Specifically, 30 mL of ink was loaded into a tempered glass container, and computer-controlled feeding delivered the ink to an ultrasonic nozzle (factory-original component, Sono-Tek), with dry, oil-free air at 551.6 kPa (80 PSI) propelling the ink droplets onto the carbon substrate. The substrate was masked using a stainless-steel square stencil (6 × 6 cm^2) to define the catalyst deposition area and was heated on a hot plate at 70 °C to facilitate solvent evaporation and promote uniform spray-deposition/growth of the catalyst across the carbon substrate surface. Catalyst loading was tuned by either increasing the ink concentration or the number of spray coatings. However, an ink concentration of >1000 mg/90 mL might lead to nozzle clogging. Therefore, a standard ink formulation of 300 mg CoTAP/KB dispersed in 90 mL of solution—corresponding to 60 mg of CoTAP—was adopted to ensure stable and consistent spray performance. In this study, 10 spray passes were identified as the optimal condition for uniform electrode fabrication, the loading of CoTAP/KB is about 0.22 mg cm^{-2} (the pure CoTAP loading is 0.044 mg cm^{-2}).

S-1.4. Electrochemical CO_2 Reduction (ECR) Test:

The prepared electrode was cut and used as the working electrode in a custom three-electrode

electrolyzer, with Hg/HgO (1.0 M KOH) as the reference electrode and Pt wire as the counter electrode, the detailed components and main equipment are reported previously. The 1.0 M KOH solution served as the electrolyte. The carbon-based gas diffusion electrode (GDE) was masked with a tape ring (0.5 cm^2 hole) and used as the cathode, with CO_2 fed from the back of the GDE, and the flow controlled by a flow meter (e.g., 15 sccm). This setup optimizes the solid-liquid-gas interface and prevents electrode flooding. The anode and cathode chambers were separated by a Nafion-117 membrane, which was chosen to avoid the transfer of ECR products into the anode, as this could cause errors, even though an anion exchange membrane may offer higher exchange ratios.[2] After connecting the setup to the electrochemical workstation, gas products were collected in a gas bag, and their volume was measured using a flow meter. The gases were analyzed by GC-FID and selectivity and partial current density were obtained (details in the next section). The applied potentials were adjusted to optimize faradaic efficiency and current density for CO_2 reduction. The potential vs. reference was converted to the reversible hydrogen electrode (RHE) using the equation:

$$E\text{ (vs. RHE)} = E\text{ (vs. Ref.)} + 0.0592 \times \text{pH} + E_0\text{ (Ref.)} - iR$$

Where E (vs. Ref.) is the applied potential, E_0 (Ref.) is the standard potential of the reference electrode (0.105 V for Hg/HgO in 1.0 M KOH),[3] and iR is the ohmic drop.

For standard ECR tests, electrolysis was performed on an MFK-A GDE for about 900 seconds using 25 mL KOH electrolyte in both the cathode and anode chambers. For long-term durability tests, liquid pumps were used to refresh the anolyte and catholyte, and ABOUND-GDL was employed to prevent flooding.

S-1.5. Qualitative and Quantitative Analysis of ECR Products:

In this work, we use gas chromatography with a flame ionization detector (GC-FID) to analyze the ECR products. Gases displayed different peak positions in real-time based on molar mass, with lighter gases appearing earlier and heavier gases later in the intensity vs. time pattern. The main ECR products included H_2, CH_4, CO, and C_2H_4. The first three gases were detected using a thermal conductivity detector (TCD), while C_2H_4 was detected only by FID, likely due

to FID's sensitivity to hydrocarbons. Following the principles of GC, H_2, CH_4, and CO eluted in sequence with retention times around 0.7, 3.0, and 5.9 minutes, respectively. Slight variations in peak times occurred due to carrier gas flow rates but did not affect the overall peak order, enabling reliable qualitative analysis.

Peak area in the intensity vs. time graph is proportional to gas concentration. Calibration lines were created for four gases by preparing standard mixtures. For example, a 20% CO standard was made by mixing 80 mL of CO_2 with 20 mL of CO in a sample bag and analyzing it with GC-FID. Each sample was measured three times for accuracy. Calibration curves showed R^2 values over 0.99, indicating high reliability.

During ECR electrolysis, gas products were collected in sample bags, and total volume was recorded. Gas content and peak areas were analyzed with GC-FID, and gas ratios were determined using the calibration curves. The detailed ECR system and electrolyzer are shown in **Scheme S1**. From this, the molar quantities of each gas were calculated. For example, CO moles (n_{CO}) were determined from peak areas. Using the CO_2-to-CO reaction stoichiometry, the electron transfer for CO production was calculated with $Q_{CO} = n_{CO} \times F \times N_{CO}$, where F is Faraday's constant (96485 C/mol), and N_{CO} is the number of electrons transferred ($N_{CO} = 2$). The chemical reactions and corresponding numbers of electrons transferred for each reaction are shown in **Table S2**.[4,5]

The total charge (Q_{total}) was determined by $Q_{total} = I \times t$, where I is the current recorded by the electrochemical workstation, and t is the relative time. Faradaic efficiency for CO (FE_{CO}) was calculated as $FE_{CO} = Q_{CO} / Q_{total}$. Finally, the total current density (J_{total}) was calculated by dividing the current by the electrode surface area (0.5 cm^2), and the partial current density for CO (J_{CO}) was determined using $J_{CO} = J_{total} \times FE_{CO}$.

S-1.6. Computational methods：

In this study, we performed density functional theory (DFT) calculations using the Vienna Ab initio Simulation Package (VASP) to investigate the electronic and magnetic properties of the system. The generalized gradient approximation (GGA) with the RPBE functional was

employed to describe the exchange-correlation effects.[6–8] The plane-wave basis set energy cutoff (ENCUT) was set to 520 eV to ensure accurate results. Spin-polarized calculations were performed, allowing for the consideration of magnetic effects. The electronic convergence criterion was set to 1×10^{-6} eV, while the convergence criterion for ionic relaxation was set to −0.05 eV/Å. We utilized the DFT-D3 method with Becke-Johnson damping to account for van der Waals interactions, ensuring that dispersion forces were included.[8,9]

Adsorbate binding and free energies were calculated with the lowest energy adsorbate conFigureuration. The binding energies of CO and COOH are, respectively,

$$E_b(CO) = E(*CO) + \Delta E_{sol}(CO) - E* - E(CO_2) - E(H_2) + E(H_2O)$$

$$E_b(COOH) = E(*COOH) + \Delta E_{sol}(COOH) - E* - E(CO_2) - \frac{1}{2}E(H_2)$$

Where ΔE_{sol} indicates a solvation correction to the calculated adsorbate energy. ΔE_{sol}(COOH)=−0.25 eV and ΔE_{sol}(CO) = −0.1 eV.[10] The free energies (G) of the adsorbate states were calculated by incorporating zero-point energies, entropies, and heat capacities, as outlined in the reference.[10] The activity volcano plots were developed by our previous work.[11] All the multilayered CoPc structures are available in the GitHub https://github.com/tohokudizhang/CoPc_ChargeTransfer.

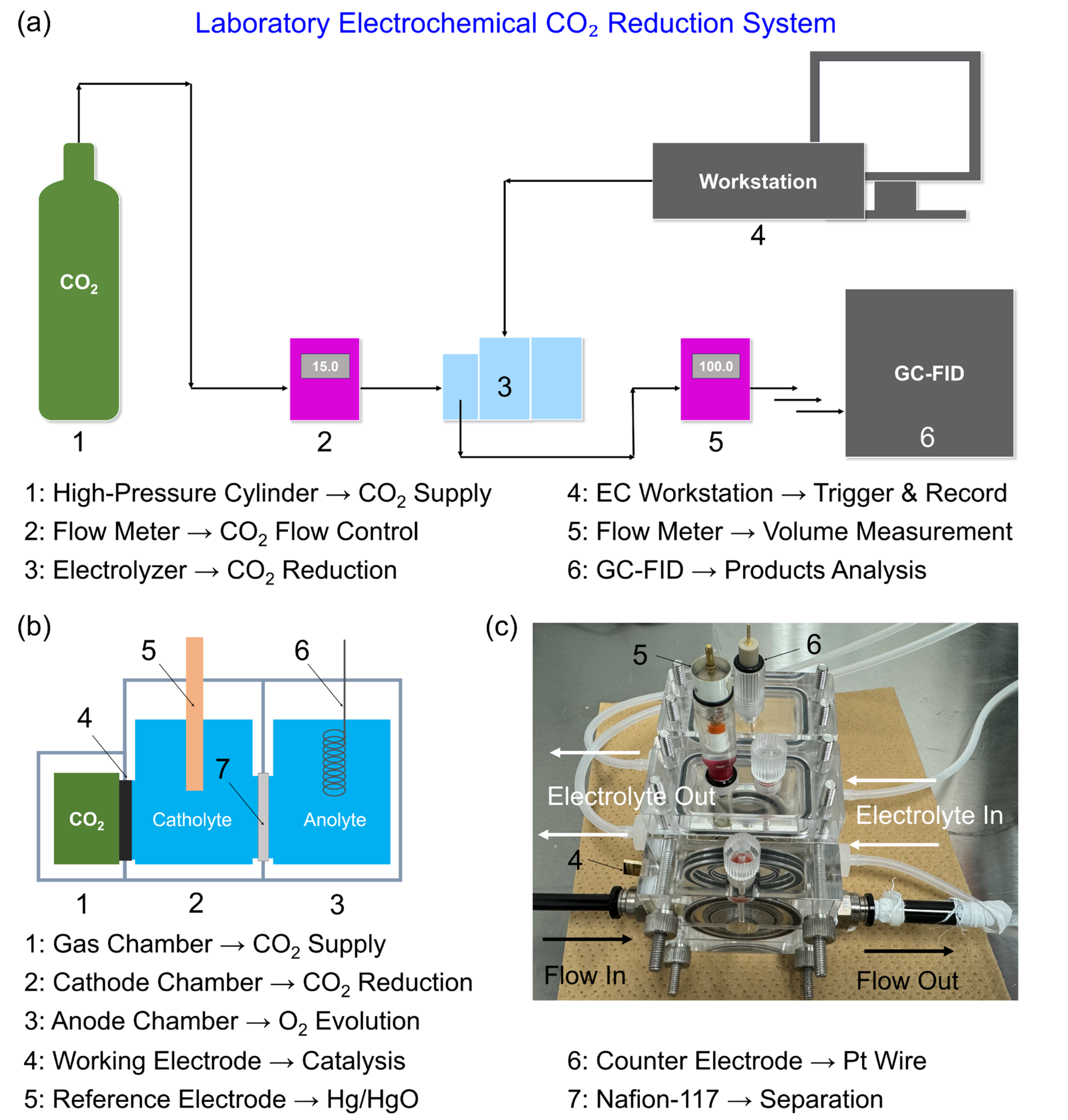

Scheme S1. Laboratory electrochemical CO_2 reduction (ECR) system: (a) main components and equipment of the laboratory ECR system; (b) schematic diagram, and (c) actual image of the custom-made three-electrode electrolyzer.

Table S1. Chemical reactions and numbers of electrons transferred for each reaction.[4,5]

Product	N	Half Reaction (pH =14)	E_0 (vs. RHE)
**H_2**	2	$2H_2O$ (l) + $2e^-$ → H_2 (g) + $2OH^-$	0
**CO**	2	CO_2 (g) + H_2O (l) + $2e^-$ → CO (g) + $2OH^-$	-0.106
**CH_4**	8	CO_2 (g) + $6H_2O$ (l) + $8e^-$ → CH_4 (g) + $8OH^-$	0.164
**C_2H_4**	12	$2CO_2$ (g) + $8H_2O$ (l) + $12e^-$ → C_2H_4 (g) + $12OH^-$	0.074

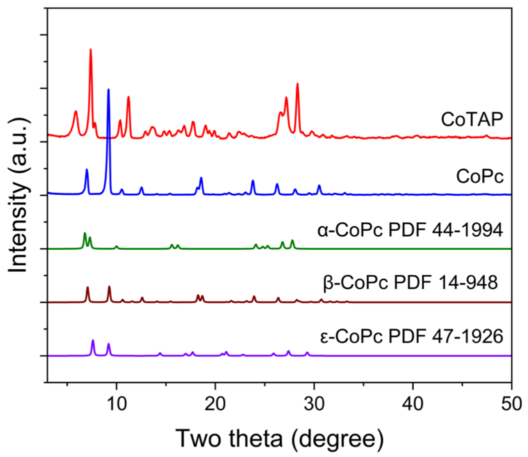

Figure S1. XRD patterns of CoTAP, CoPc and standard references.

Note: The CoPc powder used in this study shows excellent agreement with the standard β-phase cobalt phthalocyanine (β-CoPc), consistent with our previous findings.[12] In contrast, CoTAP exhibits significantly different diffraction peaks in both position and intensity. These variations indicate that the incorporation of pyridinic-N atoms, which possess additional lone-pair electrons, perturbs the intermolecular π–π stacking and modifies the electron density distribution. As a result, the crystal structure becomes distorted and the molecular packing is altered, leading to broader peaks and intensity changes in the XRD pattern.

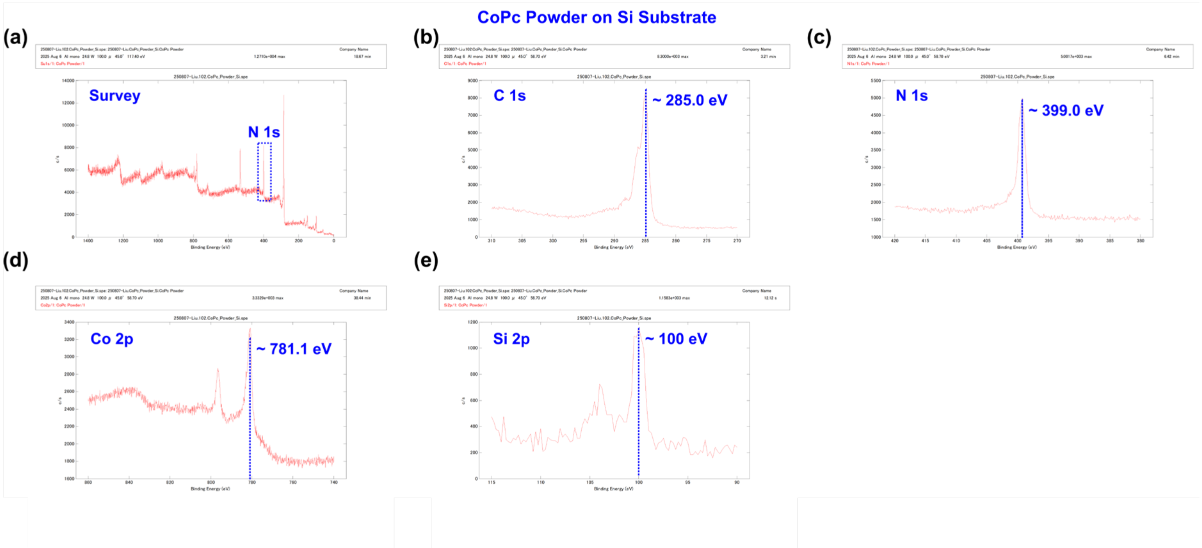

Figure S2. Unprocessed XPS spectra of CoPc powder on a Si substrate, as obtained directly from the XPS instrument: (**a**) survey spectrum, (**b**) C 1s, (**c**) N 1s, (**d**) Co 2p, and (**e**) Si 2p spectra.

Note: The survey spectra clearly indicate a significantly higher nitrogen content in CoTAP (**Figure S3** in this supporting information) than in CoPc, as reflected by the relative peak intensities—consistent with their chemical structures and confirming the successful incorporation of N atoms into the CoPc backbone (**Figure S2a** and **S3a**). Moreover, in the high-resolution spectra for C 1s, N 1s, and Co 2p, CoTAP exhibits obvious binding energy shifts of ~2.5 eV relative to CoPc, while the Si 2p peaks remain unchanged **(Figure S2b-e** and **S3b-e).** This confirms that the observed shifts originate from structural differences—specifically, the electron-donating effect of pyridinic N—rather than from instrumental or calibration errors.

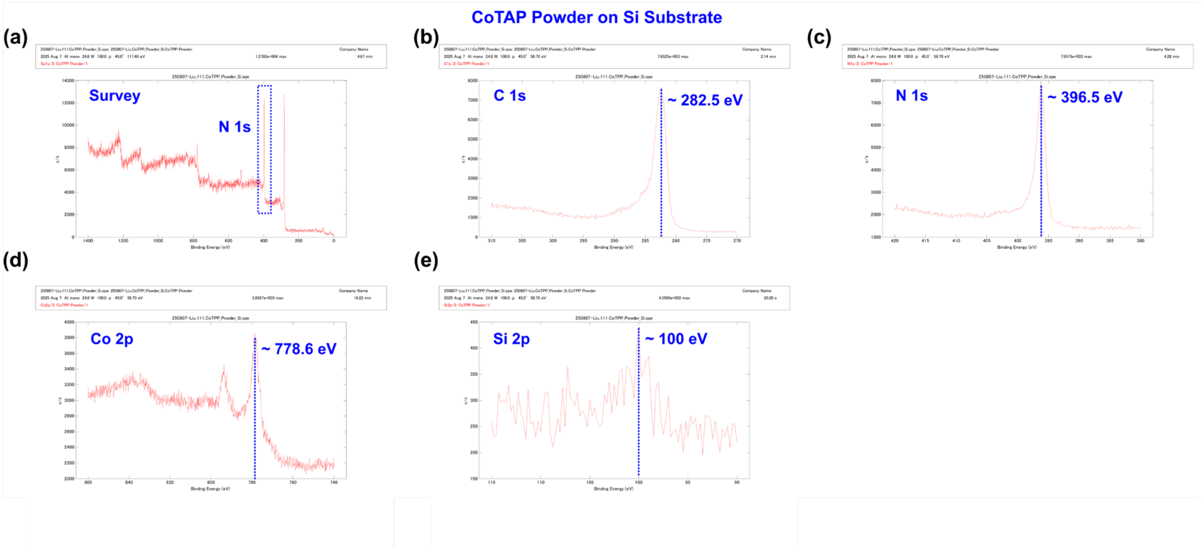

Figure S3. Unprocessed XPS spectra of CoTAP powder on a Si substrate, as obtained directly from the XPS instrument: (**a**) survey spectrum, (**b**) C 1s, (**c**) N 1s, (**d**) Co 2p, and (**e**) Si 2p spectra.

Note: The survey spectra clearly indicate a significantly higher nitrogen content in CoTAP than in CoPc (**Figure S2** in this supporting information), as reflected by the relative peak intensities—consistent with their chemical structures and confirming the successful incorporation of N atoms into the CoPc backbone (**Figure S2a** and **S3a**). Moreover, in the high-resolution spectra for C 1s, N 1s, and Co 2p, CoTAP exhibits obvious binding energy shifts of ~2.5 eV relative to CoPc, while the Si 2p peaks remain unchanged **(Figure S2b-e** and **S3b-e).** This confirms that the observed shifts originate from structural differences—specifically, the electron-donating effect of pyridinic N—rather than from instrumental or calibration errors.

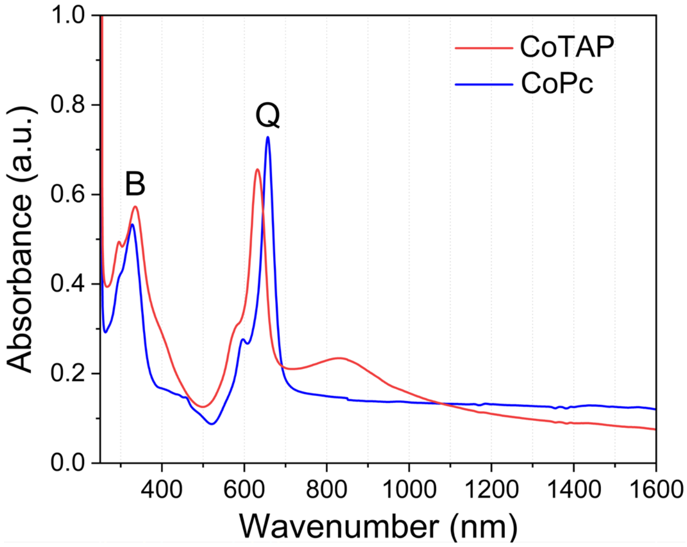

Figure S4. UV-vis-NIR spectra of CoTAP and CoPc.

Note: A quartz cuvette containing 285 μL of DMSO was first measured as the background reference. Subsequently, 15 μL of CoPc or CoTAP ink (prepared at a concentration of 1.1 mg catalyst per 5 mL DMSO) was introduced into the cuvette for UV-vis measurements. The background-corrected spectra reveal that, compared with CoPc, CoTAP exhibits a clear red shift in the B band (~370 nm) and a blue shift in the Q band (550-700 nm). More importantly, CoTAP displays a unique absorption feature in the 720-900 nm region, which may originate from electronic density redistribution induced by the incorporation of pyridinic-N or from charge-transfer (CT) transitions. These results provide strong evidence that pyridinic-N has been successfully incorporated into the CoPc backbone, consistent with the preceding analytical findings.

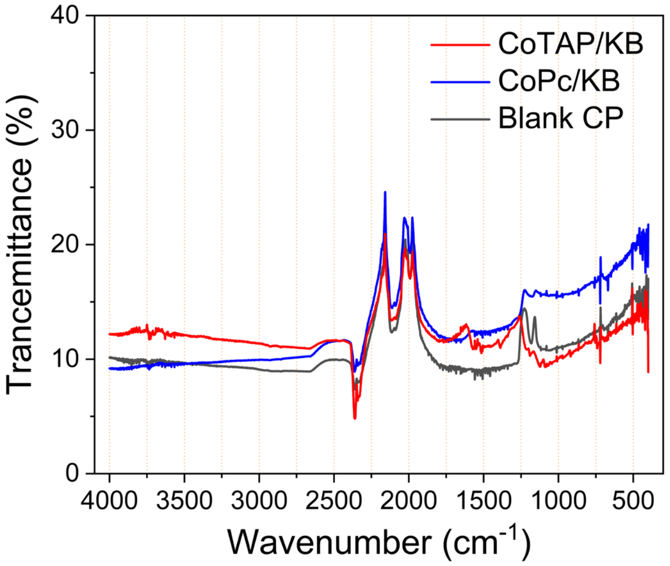

Figure S5. Typical FTIR patterns of blank carbon paper, CoTAP/KB hybrid, CoPc/KB hybrid electrodes.

Note: The blank carbon paper (CP) exhibits two pairs of shoulder peaks corresponding to characteristic C–C/C=C vibrations at 1000–1300 cm^{-1} and 1800–2250 cm^{-1}. In contrast, both CoTAP/KB and CoPc/KB samples retain the prominent shoulder peaks at 1800–2250 cm^{-1}, indicating that the KB preserves its graphene-like structure. Notably, these two samples display distinct peaks in the 900–1300 cm^{-1} region, attributable to Co–N and C=N species. It is worth noting that CoTAP shows a specific peak around 1600 cm^{-1}, which may correspond to pyridinic-N introduced at the β-position of the CoPc backbone. Additionally, the increased transmittance observed in the 2500–4000 cm^{-1} range can be attributed to the presence of pyridinic-N. These observations are consistent with previous MALDI, XPS, and SAXS results, providing strong evidence that Pyridinic-N has been successfully incorporated into the CoPc backbone.

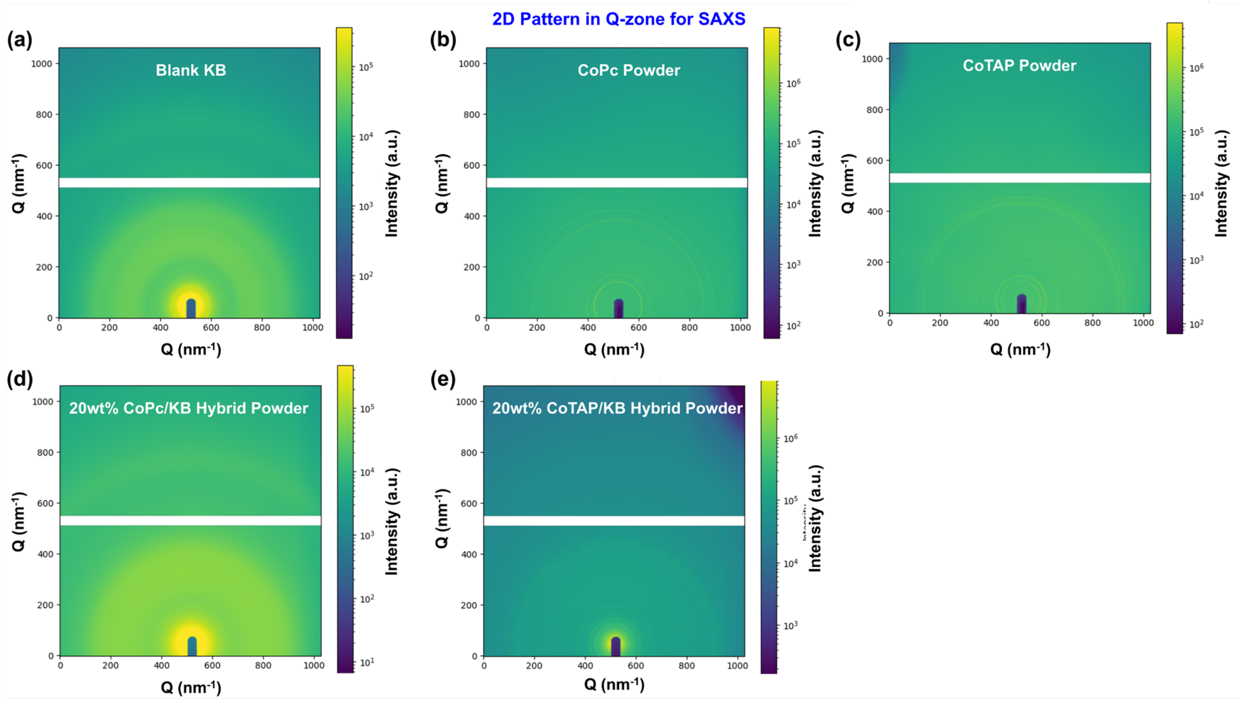

Figure S6. Typical 2D SAXS patterns (Q-zone) of (**a**) blank KB, (**b**) pristine CoPc, (**c**) CoTAP, (**d**) 20 wt% CoPc/KB hybrid, and (**e**) 20 wt% CoTAP/KB hybrid powders.

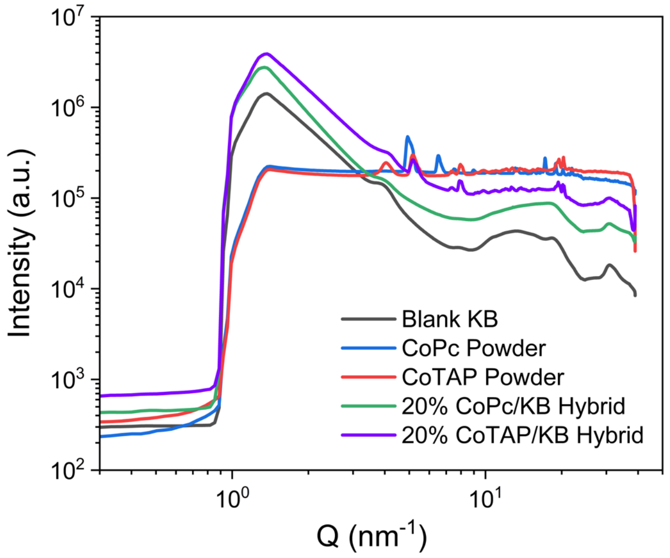

Figure S7. Typical 1D-integrated SAXS patterns of blank KB, pristine CoPc, 5 wt% CoPc/KB hybrid, 20 wt% CoPc/KB hybrid, and the 20 wt% physical mixture of CoPc and KB powders.

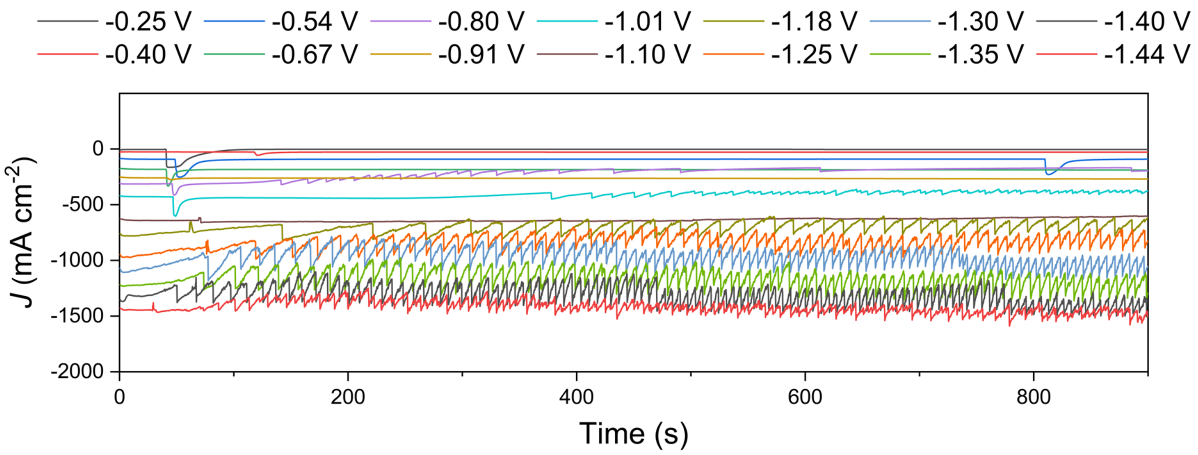

Figure S8. Time-dependent total current density curves at various applied potentials for CO_2 reduction using the CoTAP/KB electrodes in 1.0 M KOH.

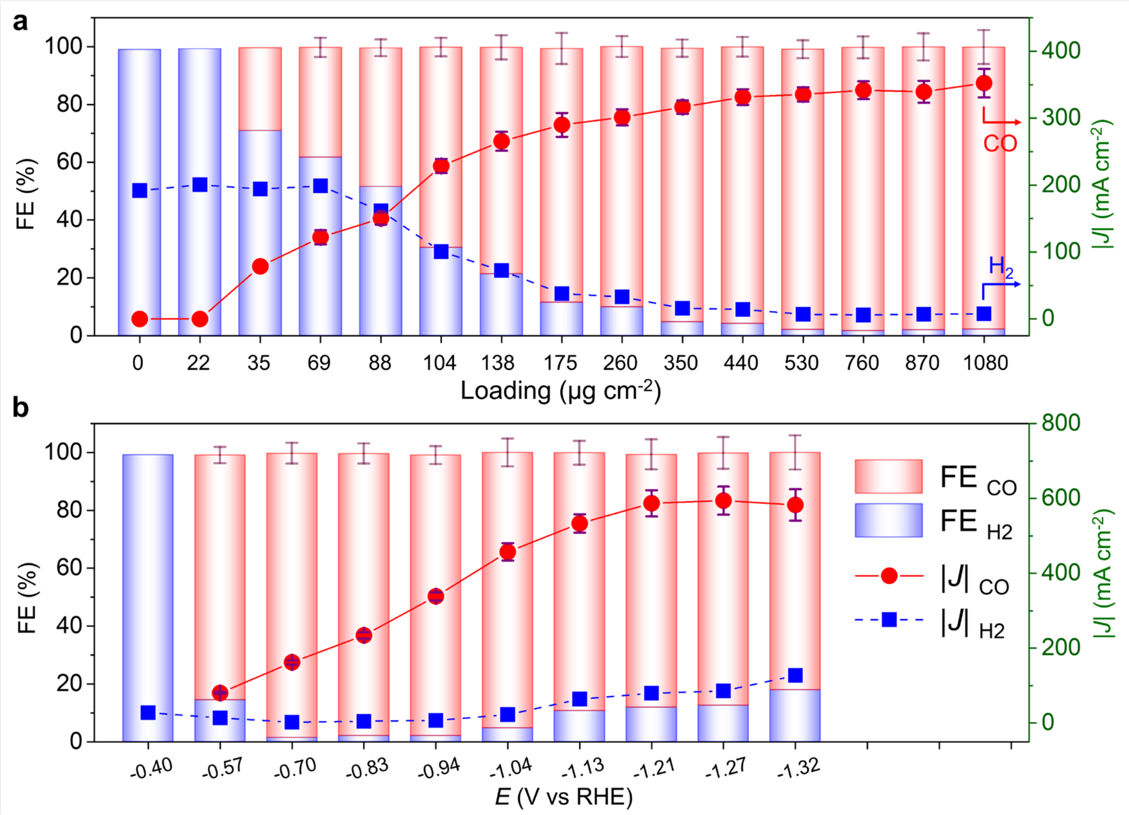

Figure S9. Electrocatalytic performance of CoPc/KB GDE for electrochemical CO2 reduction (ECR). (**a**) Faradic efficiency (FE, left axis) and absolute current density (|*J*|, right axis) for CO (red) and H_2 (blue) for electrodes at different loadings, at a potential (*E*) of -0.94V (vs. RHE). (**b**) FE (left axis) and |*J*| (right axis) as a function of applied potential (vs. RHE) at an optimized catalyst loading of ~500 μg cm^{-2}.

Note: Within the tested loading range of 100~1000 μg cm^{-2}, CO selectivity consistently exceeded 90%, with the CO current density (J_{CO}) remaining higher than -300 mA cm^{-2}. A loading of 500 μg cm^{-2} was identified as optimal for potential-dependent testing. Under these conditions, the total current density (J_{total}) increased significantly with more negative potentials, peaking at a J_{CO} of -595 mA cm^{-2} at -1.27 V vs. RHE.

Table S2. Comparation of the key-values in this work and selected literature, serving as a supplement to Table 1. The complete dataset is also accessible via the DigCat database: https://www.digcat.org/.

Catalyst	Electrolyte	FE_{CO} (%)	E_1 (V)	J_{CO} (mA cm^{-2})	E_2 (V)	Loading (mg cm^{-2})	Ref.
CoTAP/KB	1.0M KOH	>98.0	-0.25 ~ -1.40	-1084	-1.40	0.2	This work*
CoPc/KB	1.0M KOH	>98.0	-0.70 ~ -1.04	-595	-1.27	0.5	This work*
CoPc/CNT-MDE	0.5M $KHCO_3$	90.0	-1.0	-50	-1.10	-	[13]
CoPc/CNT-MDE	0.1M $KHCO_3$	98.0	-0.63	-15	-0.63	0.4	[14]
CoPc/CNT-MDE	H_2SO_4+K_2SO_4	73.0	-	-38	-	0.8	[15]
CoPc/CB-MDE	1.0M $KHCO_3$	98.0	-1.05	-100	-0.65	-	[16]
CoPc/CNT-MDE	0.1M $KHCO_3$	97.0	-	-200	-	-	[17]
CoPc/CNT-ODA	0.5M $KHCO_3$	97.7	-1.0	-350	-1.3	1.0	[18]
CoPc-TBG/CNT	1.0M KOH	96.0	-0.72	-112	-0.72	0.2	[19]
CoPc- EtO_8/CNP	1.0M $KHCO_3$	95.0	-2.20	-340	-2.20	-	[20]
CoPc-OCH_3/CNT	0.5M $KHCO_3$	97.0	-1.0	-280	-1.10	-	[13]
CoPc-NO_2/CNT	0.5M $KHCO_3$	95.0	-0.9	-80	-1.10	-	[13]
CoPPc/CNT	0.5M $KHCO_3$	90.0	-0.60	-19	-0.60	1.0	[21]
NiPc/CNT-MDE	0.5M $KHCO_3$	>98.0	-0.54 ~ -0.68	-400	-0.70	0.4	[22]
NiPc/NHCSs	0.5M $KHCO_3$	98.6	-0.87	-25	-1.05	1.0	[23]
NiPc$(OH)_6$(DCNFO)/CNT	1.0M KOH	>98.0	-0.80 ~ -1.40	-380	-1.40	-	[24]
NiPc-OMe	H_2SO_4+K_2SO_4	98.0	-1.15	-400	-1.45	1.0	[25]

Note: FE_{CO}: the maximum faradic efficiency for CO; $\boldsymbol{E_1}$: the potentials (vs RHE) of maximum FE_{CO}; $\boldsymbol{J_{CO}}$**:** the maximum current density for CO; $\boldsymbol{E_2}$: the potentials (vs RHE) of maximum J_{CO}; **CNT:** carbon nanotube; **CB:** carbon black; **MDE:** molecularly-dispersed electrocatalyst; **ODA:** octadecylamine; **TBG:** three tert-butyl groups; **CoPPc:** cobalt poly-phthalocyanine; **NHCS:** nitrogen-doped hollow carbon nanospheres.

Table S3. Comparation of the key-values in this work and selected literature for non-Pc-based catalysts.

**Catalyst**	**Electrolyte**	**FE_{CO} (%)**	**E_1 (V)**	**J_{CO} (mA cm^{-2})**	**Ref.**
CoTAP/KB	1.0M KOH	>98.0	-0.25 ~ -1.40	-1084	This work*
P-Ni-NC-50	1.0 M KOH	90.6	90.6	-244.1	[26]
AgCNCB	1.0 M KOH	96	96	-287	[27]
CuSbOx	0.5 M $KHCO_3$	81.5	81.5	-283	[28]
Ni-Mn-NC	0.5 M $KHCO_3$	99.2	99.2	-328	[29]
Ag@4.0-PPFDA	1.0 M KOH	93.5	93.5	-	[30]
Er SAC	0.5 M K_2SO_4	90.2	90.2	-	[31]
Fe-P-N-C	1.0 M KOH	96	96	580	[32]
Zn-N/Se-C	0.1 M $KHCO_3$	97.3	97.3	539.7	[33]
nano Ag	0.5 M $KHCO_3$	97.29	97.29	632.67	[34]
500-ZnO	3.0 M KCl	98.3	98.3	786.56	[35]
Zn HPE	3.0 M KCl	90.3	90.3	-	[36]
Zn HPE	3.0 M KCl	81.7	81.7	-	[36]

Note: FE_{CO}: the maximum faradic efficiency for CO; $\boldsymbol{E_1}$: the potentials (vs RHE) of maximum FE_{CO}; $\boldsymbol{J_{CO}}$**:** the maximum current density for CO.

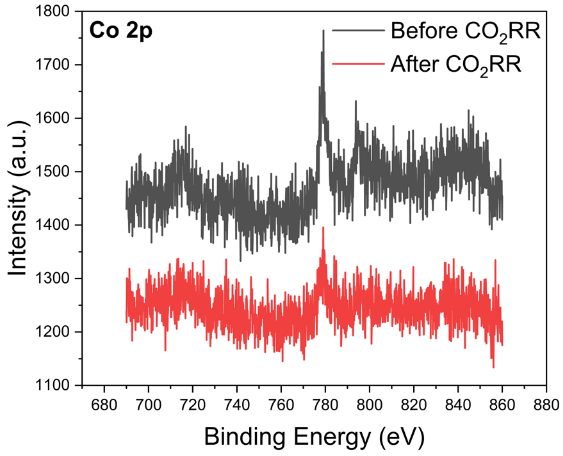

Figure S10. Co 2p spectra of the CoTAP/KB electrode before and after long-term CO_2 reduction electrolysis.

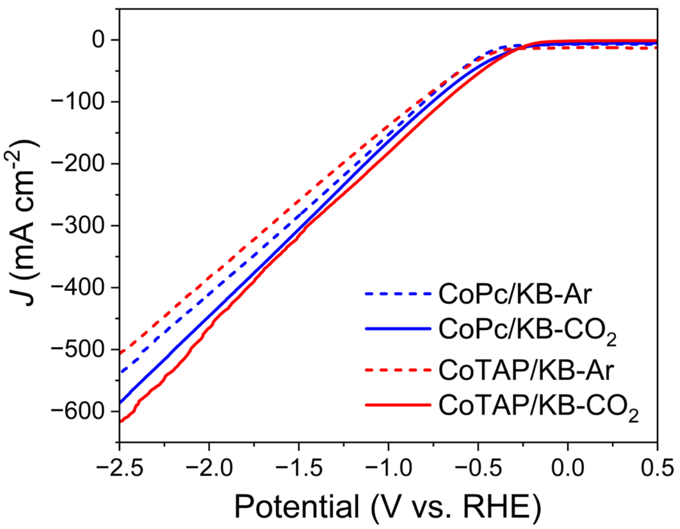

Figure S11. LSV curves of CoTAP/KB and CoPc/KB GDEs under CO_2 and Ar atmospheres without iR compensation.

Note: To preliminarily evaluate the electrocatalytic response under CO_2 and Ar, comparative control experiments were conducted. As shown in the LSV data, both CoTAP/KB and CoPc/KB exhibit higher current densities under CO_2 than under Ar, reflecting differences in reaction rates. Notably, CoTAP/KB displays higher current densities than CoPc/KB, indicating enhanced catalytic activity due to the incorporation of pyridinic-N, which is consistent with the electrocatalytic performance discussed in the main manuscript.

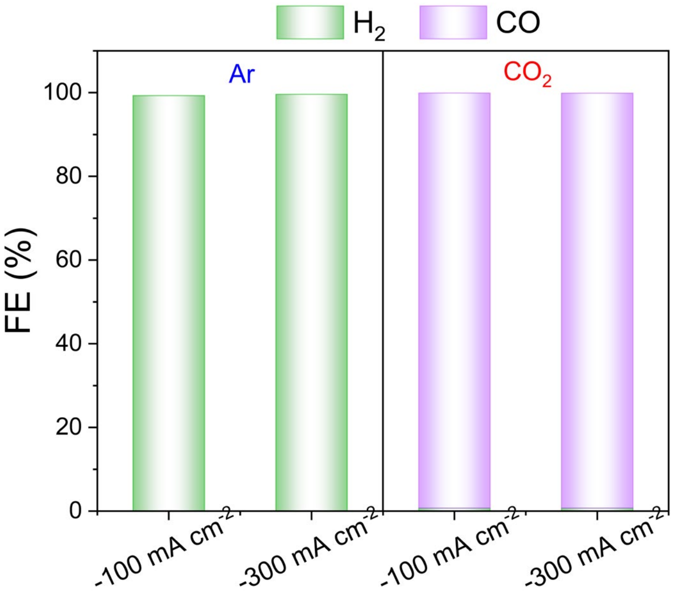

Figure S12. FEs of CoTAP/KB GDEs under CO_2 and Ar atmospheres at current densities of -100 and -300 mA cm^{-2}.

Note: To preliminarily verify the carbon source of CO in our system, we have carried out comparative control experiments under CO_2 and Ar atmospheres. Gas products were analyzed using GC-FID. Under a CO_2 atmosphere, over 98% CO selectivity was observed, while under Ar, only H_2 was detected. All other conditions were kept constant, and the only variable was the gas atmosphere, thereby confirming that the CO originates from the supplied CO_2.

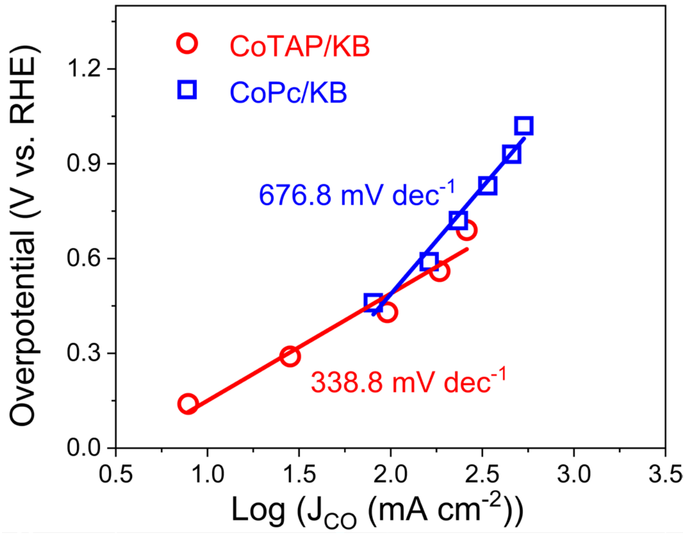

Figure S13. Tafel slopes of CoTAP/KB and CoPc/KB electrodes in 1 M KOH solution.

Note: The results show that the smaller Tafel slope observed for CoTAP compared to CoPc, indicating faster reaction kinetics in the ECR process. A lower Tafel slope generally implies that the rate-determining step involves a reduced activation barrier, thereby enabling more efficient electron and proton transfer. This enhancement can be attributed to the incorporation of pyridinic nitrogen in CoTAP, which optimizes the electronic environment of the active Co-N sites, facilitates CO_2 adsorption and activation, and ultimately accelerates the overall catalytic process.

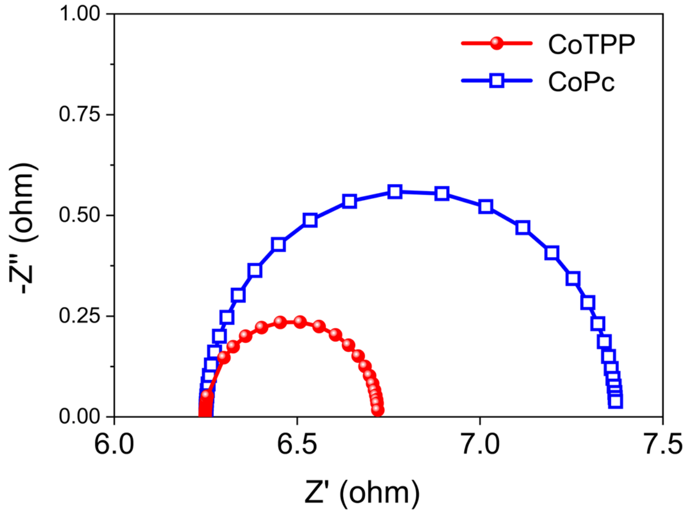

Figure S14. The in situ electrochemical impedance spectroscopy (EIS) measurements of CoTAP/KB and CoPc/KB electrodes at -0.91 V vs. RHE in 1 M KOH solution.

Note: The *in-situ* EIS measurements were conducted on CoTAP/KB and CoPc/KB electrodes under a CO_2 atmosphere at the same applied potential. The measured charge transfer resistance (R_{ct}) values were 0.4 and 1.1 Ω cm^{-2}, respectively, indicating a substantial decrease in R_{ct} for CoTAP. This result highlights the markedly lower resistance and superior charge transfer efficiency of the CoTAP system compared to CoPc.

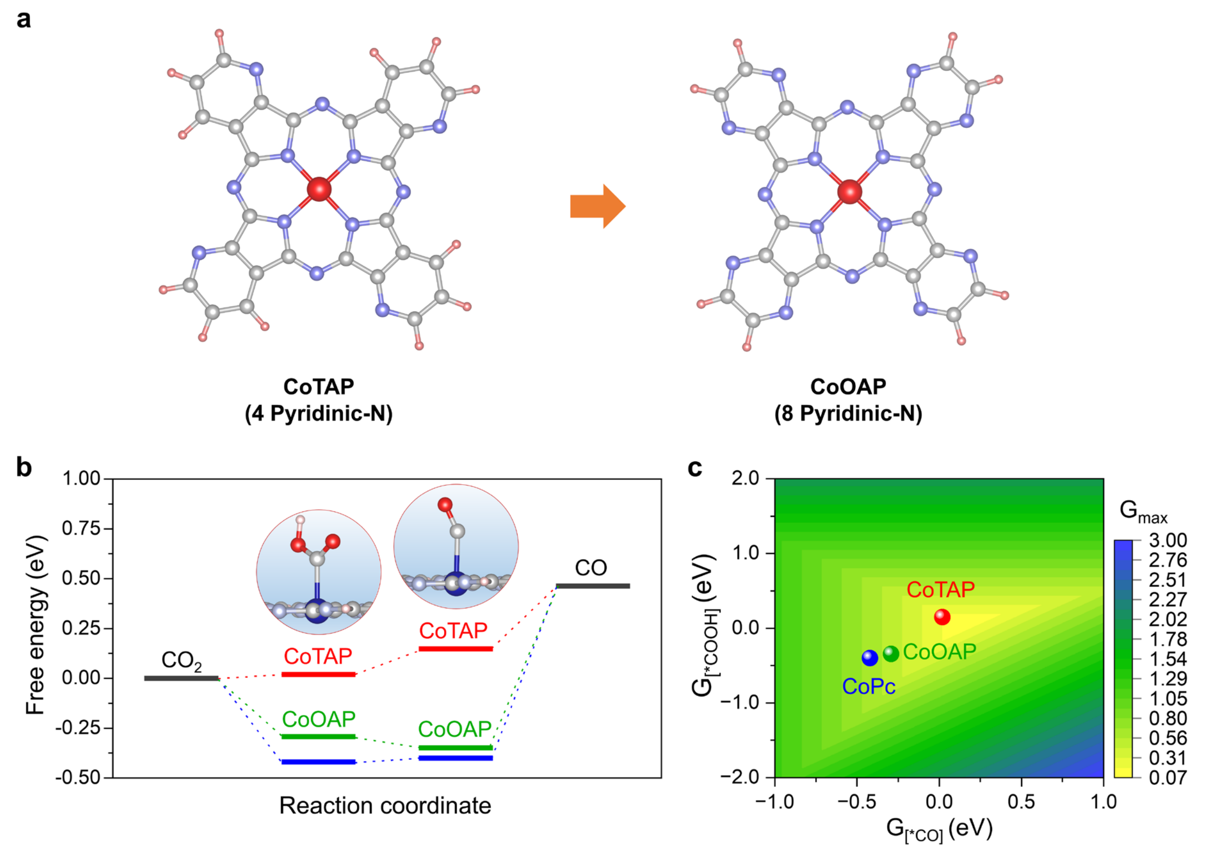

Figure S15. Comparison of free energies for CoTAP and CoOAP structures: (**a**) Molecular structures of CoTAP and Cobalt Octa-Aza-Phthalocyanine (CoOAP), **(b)** DFT-calculated adsorption free energies ($G_{[*COOH]}$ and $G_{[*CO]}$) for CoTAP/C and CoOAP/C structures. **(c)** Volcano model of ECR as the function of $G_{[*COOH]}$ and $G_{[*CO]}$, illustrating the intrinsic activity enhancement of CoTAP and CoOAP.

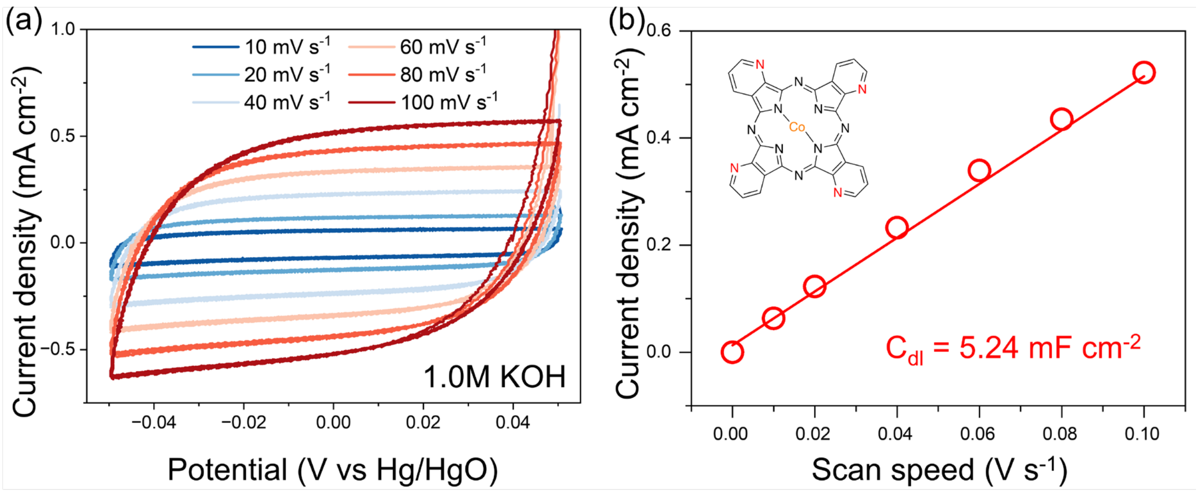

Figure S16. ECSA measurement of CoTAP/KB electrodes. (**a**) Cyclic voltammetry (CV) curves of CoTAP/KB electrodes in 1 M KOH solution at various scan rates, ranging from -0.05 to 0.05 V vs. Hg/HgO. (**b**) Double-layer capacitance (C_{dl}) derived from fitting the capacitance current density (j_C) against the scan rate (v) for CoTAP/KB/CP electrodes.

Note: CV scans were performed on CoTAP/KB to calculate the C_{dl} at varying scan rates, allowing for the determination of the ECSA.[2,37]The results indicate that the C_{dl} value for CoTAP/KB is 5.24 mF cm^{-2}, corresponding to an ECSA of 131.0 cm^2 cm^{-2}. These findings clearly demonstrate that the incorporation of KB significantly increases the ECSA. While ECSA primarily reflects the solid-liquid interface, previous studies suggest that a higher ECSA enhances the three-phase boundary, which facilitates the exposure of more active sites for CO_2RR.

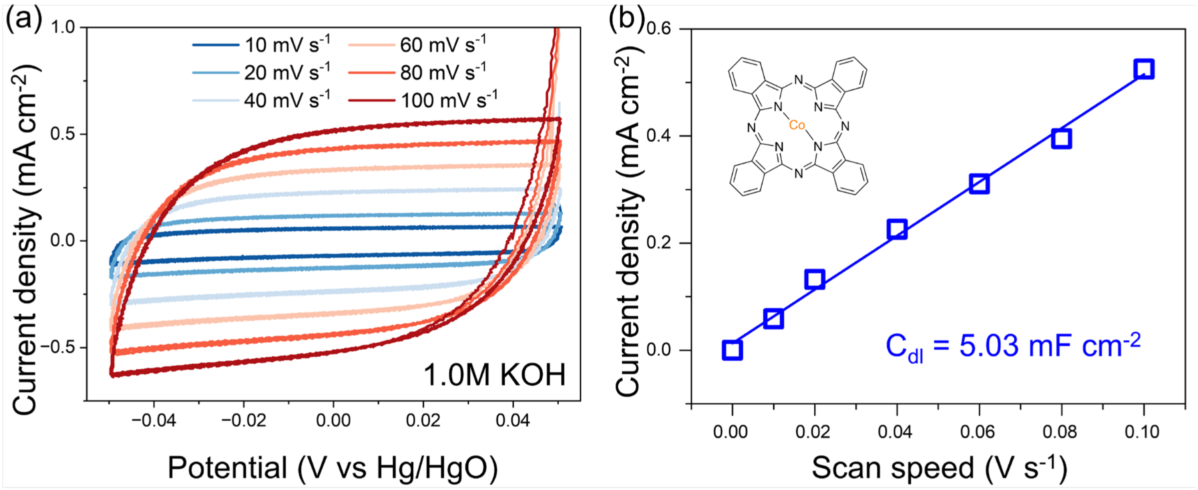

Figure S17. ECSA measurement of CoPc/KB electrodes. (**a**) Cyclic voltammetry (CV) curves of CoPc/KB electrodes in 1 M KOH solution at various scan rates, ranging from -0.05 to 0.05 V vs. Hg/HgO. (**b**) Double-layer capacitance (C_{dl}) derived from fitting the capacitance current density (j_C) against the scan rate (v) for CoPc/KB/CP electrodes.

Note: The results indicate that the C_{dl} value for CoPc/KB is 5.03 mF cm^{-2}, corresponding to an ECSA of 125.8 cm^2 cm^{-2}. These findings suggest that CoTAP and CoPc possess similar ECSAs; however, their CO_2RR performances differ significantly. CoTAP achieves a maximum $|J|_{CO}$ of 1084 mA cm^{-2} (geometric area), compared to 595 mA cm^{-2} for CoPc. When normalized by ECSA, CoTAP delivers an intrinsic current density of 8.7 mA unit^{-1}, nearly double that of CoPc (4.5 mA unit^{-1}). Collectively, these results highlight that the superior electrocatalytic performance of CoTAP primarily arises from its enhanced intrinsic activity at the molecular active sites.

Reference in the Supporting Information:

[1] H. Abe, Y. Hirai, S. Ikeda, Y. Matsuo, H. Matsuyama, J. Nakamura, T. Matsue, H. Yabu, *NPG Asia Mater.* **2019**, *11*, 57.

[2] T. Liu, K. Ohashi, K. Nagita, T. Harada, S. Nakanishi, K. Kamiya, *Small* **2022**, *18*, 2205323.

[3] K. Kawashima, R. A. Márquez, Y. J. Son, C. Guo, R. R. Vaidyula, L. A. Smith, C. E. Chukwuneke, C. B. Mullins, *ACS Catal.* **2023**, *13*, 1893.

[4] Z. Yue, C. Ou, N. Ding, L. Tao, J. Zhao, J. Chen, *ChemCatChem* **2020**, *12*, 6103.

[5] T. Liu, H. Yabu, *EcoEnergy* **2024**, *2*, 419.

[6] B. Hammer, L. B. Hansen, J. K. Nørskov, *Phys. Rev. B - Condens. Matter Mater. Phys.* **1999**, *59*, 7413.

[7] J. Y. Jung, J. H. Park, Y. J. Jeong, K. H. Yang, N. K. Choi, S. H. Kim, W. J. Kim, *Korean J. Physiol. Pharmacol.* **2006**, *10*, 289.

[8] K. Li, L. Luo, Y. Zhang, W. Li, Y. Hou, *ACS Appl. Mater. Interfaces* **2018**, *10*, 41525.

[9] A. Allouche, *J. Comput. Chem.* **2012**, *32*, 174.

[10] K. Chan, C. Tsai, H. A. Hansen, J. K. Nørskov, *ChemCatChem* **2014**, *6*, 1899.

[11] W. Yang, Z. Jia, B. Zhou, L. Chen, X. Ding, L. Jiao, H. Zheng, Z. Gao, Q. Wang, H. Li, *ACS Catal.* **2023**, *13*, 9695.

[12] T. Liu, D. Zhang, Y. Hirai, K. Ito, K. Ishibashi, N. Todoroki, Y. Matsuo, J. Yoshida, S. Ono, H. Li, H. Yabu, *Adv. Sci.* **2025**, *12*, 202501459.

[13] M. Huang, B. Chen, H. Zhang, Y. Jin, Q. Zhi, T. Yang, K. Wang, J. Jiang, *Small Methods* **2024**, *4*, 2301652.

[14] X. Zhang, Z. Wu, X. Zhang, L. Li, Y. Li, H. Xu, X. Li, X. Yu, Z. Zhang, Y. Liang, H. Wang, *Nat. Commun.* **2017**, *8*, 14675.

[15] S. Feng, X. Wang, D. Cheng, Y. Luo, M. Shen, J. Wang, W. Zhao, S. Fang, H. Zheng, L. Ji, X. Zhang, W. Xu, Y. Liang, P. Sautet, J. Zhu, *Angew. Chemie Int. Ed.* **2024**, *63*, e202317942.

[16] M. Wang, A. Loiudice, V. Okatenko, I. D. Sharp, R. Buonsanti, *Chem. Sci.* **2023**, *14*, 1097.

[17] X. Wu, J. W. Sun, P. F. Liu, J. Y. Zhao, Y. Liu, L. Guo, S. Dai, H. G. Yang, H. Zhao, *Adv. Funct. Mater.* **2022**, *32*, 2107301.

[18] L. Xiong, X. Fu, Y. Zhou, P. Nian, Z. Wang, Q. Yue, *ACS Catal.* **2023**, *13*, 6652.

[19] M. Wang, K. Torbensen, D. Salvatore, S. Ren, D. Joulié, F. Dumoulin, D. Mendoza, B. Lassalle-Kaiser, U. Işci, C. P. Berlinguette, M. Robert, *Nat. Commun.* **2019**, *10*, 3602.

[20] S. Ren, E. W. Lees, C. Hunt, A. Jewlal, Y. Kim, Z. Zhang, B. A. W. Mowbray, A. G. Fink, L. Melo, E. R. Grant, C. P. Berlinguette, *J. Am. Chem. Soc.* **2023**, *145*, 4414.

[21] N. Han, Y. Wang, L. Ma, J. Wen, J. Li, H. Zheng, K. Nie, X. Wang, F. Zhao, Y. Li, J. Fan, J. Zhong, T. Wu, D. J. Miller, J. Lu, S. T. Lee, Y. Li, *Chem* **2017**, *3*, 652.

[22] X. Zhang, Y. Wang, M. Gu, M. Wang, Z. Zhang, W. Pan, Z. Jiang, H. Zheng, M. Lucero, H. Wang, G. E. Sterbinsky, Q. Ma, Y. G. Wang, Z. Feng, J. Li, H. Dai, Y. Liang, *Nat. Energy* **2020**, *5*, 684.

[23] S. Gong, W. Wang, R. Lu, M. Zhu, H. Wang, Y. Zhang, J. Xie, C. Wu, J. Liu, M. Li, S.

Shao, G. Zhu, X. Lv, *Appl. Catal. B Environ.* **2022**, *318*, 121813.
[24] Y. Jin, X. Zhan, Y. Zheng, H. Wang, X. Liu, B. Yu, X. Ding, T. Zheng, K. Wang, D. Qi, J. Jiang, *Appl. Catal. B Environ.* **2023**, *327*, 122446.
[25] Z. Jiang, Z. Zhang, H. Li, Y. Tang, Y. Yuan, J. Zao, H. Zheng, Y. Liang, *Adv. Energy Mater.* **2023**, *13*, 2203603.
[26] J. Sun, K. Li, Z. Liu, J. Xu, P. Gao, M. Wang, Y. Li, R. Zhu, I. P. Parkin, Z. Huang, *J. Catal.* **2025**, *445*, 116020.
[27] J. Hong, Y. E. Jeon, J. Park, Y. E. Kim, Y. N. Ko, *Appl. Surf. Sci.* **2025**, *696*, 162892.
[28] H. Q. Fu, T. Yu, J. White, J. W. Sun, Y. Wu, W. J. Li, N. M. Bedford, Y. Wang, T. E. Rufford, C. Lian, P. Liu, H. G. Yang, H. Zhao, *Chem* **2025**, *11*, 1.
[29] H. L. Guo, Y. H. Liu, L. X. Wang, N. Y. Wang, X. J. Jiang, J. Y. Pang, D. Bin Dang, X. Y. Ji, Y. Bai, *J. Colloid Interface Sci.* **2025**, *683*, 1041.
[30] Q. Chang, G. Zhang, Y. Wang, Y. Lin, H. Gao, L. Guo, P. Zhang, C. Pei, T. Wang, J. Gong, *Adv. Funct. Mater.* **2025**, *35*, 202425601.
[31] Q. Wang, T. Luo, X. Cao, Y. Gong, Y. Liu, Y. Xiao, H. Li, F. Gröbmeyer, Y. R. Lu, T. S. Chan, C. Ma, K. Liu, J. Fu, S. Zhang, C. Liu, Z. Lin, L. Chai, E. Cortes, M. Liu, *Nat. Commun.* **2025**, *16*, 2985.
[32] Y. Zang, Y. Liu, R. Lu, Q. Yang, B. Wang, M. Zhang, Y. Mao, Z. Wang, Y. Lum, *Adv. Mater.* **2025**, *37*, 2417034.
[33] J. Chen, C. Hu, Y. Liu, Y. Wei, K. Shen, L. Chen, Y. Li, *Angew. Chemie Int. Ed.* **2025**, *64*, e202422775.
[34] L. Ma, Y. Wang, M. Zhang, J. Li, L. Zhang, X. Zhu, Q. Fu, Q. Liao, *J. Power Sources* **2025**, *641*, 236874.
[35] Z. Ling, Y. Yin, X. Kang, X. Li, R. Duan, S. Zhou, H. Liu, G. Mo, Z. Chen, X. Wu, R. Feng, Z. Wu, B. Han, X. Xing, *Chem Catal.* **2025**, *5*, 101192.
[36] X. Liu, S. Li, A. Chen, X. Dong, J. Mao, C. Zhu, G. Wu, Y. Wei, J. Xia, H. Zhu, X. Wang, Z. Xu, G. Li, Y. Song, W. Wei, W. Chen, *ACS Catal.* **2025**, *15*, 4259.
[37] T. Liu, P. Diao, Z. Lin, H. Wang, *Nano Energy* **2020**, *74*, 104787.
